# Autologous iPSC- and MSC-derived chondrocyte implants for cartilage repair in a miniature pig model

**DOI:** 10.1186/s13287-025-04215-7

**Published:** 2025-02-23

**Authors:** Ming-Song Lee, Eric Chang-Yi Lin, Athillesh Sivapatham, Ellen M. Leiferman, Hongli Jiao, Yan Lu, Brett W. Nemke, Matthew Leiferman, Mark D. Markel, Wan-Ju Li

**Affiliations:** 1 Musculoskeletal Biology and Regenerative Medicine Laboratory, Department of Orthopedics and Rehabilitation, University of Wisconsin-Madison, Madison, Wisconsin, 53705 USA; 2 Department of Biomedical Engineering, University of Wisconsin-Madison, Madison, Wisconsin, 53705 USA; 3 School of Veterinary Medicine, University of Wisconsin-Madison, Madison, Wisconsin, 53705 USA

## Abstract

**Background:**

Induced pluripotent stem cell (iPSC)-derived mesenchymal stem cells (iMSCs) have greater potential for generating chondrocytes without hypertrophic and fibrotic phenotypes compared to bone marrow-derived mesenchymal stem/stromal cells (BMSCs). However, there is a lack of research demonstrating the use of autologous iMSCs for repairing articular chondral lesions in large animal models. In this study, we aimed to evaluate the effectiveness of autologous miniature pig (minipig) iMSC-chondrocyte (iMSC-Ch)-laden implants in comparison to autologous BMSC-chondrocyte (BMSC-Ch)-laden implants for cartilage repair in porcine femoral condyles.

**Methods:**

iMSCs and BMSCs were seeded into fibrin glue/nanofiber constructs and cultured with chondrogenic induction media for 7 days before implantation. To assess the regenerative capacity of the cells, 19 skeletally mature Yucatan minipigs were randomly divided into microfracture control, acellular scaffold, iMSC, and BMSC subgroups. A cylindrical defect measuring 7 mm in diameter and 0.6 mm in depth was created on the articular cartilage surface without violating the subchondral bone. The defects were then left untreated or treated with acellular or cellular implants.

**Results:**

Both cellular implant-treated groups exhibited enhanced joint repair compared to the microfracture and acellular control groups. Immunofluorescence analysis yielded significant findings, showing that cartilage treated with iMSC-Ch implants exhibited higher expression of COL2A1 and minimal to no expression of COL1A1 and COL10A1, in contrast to the BMSC-Ch-treated group. This indicates that the iMSC-Ch implants generated more hyaline cartilage-like tissue compared to the BMSC-Ch implants.

**Conclusions:**

Our findings contribute to filling the knowledge gap regarding the use of autologous iPSC derivatives for cartilage repair in a translational animal model. Moreover, these results highlight their potential as a safe and effective therapeutic strategy.

**Supplementary Information:**

The online version contains supplementary material available at 10.1186/s13287-025-04215-7.

## Introduction

Various types of stem cells have been explored for articular cartilage regeneration [1]. For instance, bone marrow stromal cells (BMSCs) have been extensively studied for their ability to promote cartilage repair due to their chondrogenic capacity and ease of harvesting from patients. The microfracture procedure, which harnesses the regenerative capacity of endogenous BMSCs, is clinically used for cartilage lesion repair [2]. This procedure induces tissue repair by exposing subchondral BMSCs at the joint surface, facilitating cartilage regeneration. However, the repaired tissue primarily consists of fibrocartilage with a high ratio of collagen type 1 to collagen type 2 and a low amount of proteoglycans [3, 4]. Such tissue composition results in inferior mechanical properties for load resistance of the joint [5]. Additionally, the use of BMSCs for articular cartilage repair raises concerns about osteochondral ossification of the regenerated tissue. Research evidence has shown that BMSC-derived chondrocytes express hypertrophic chondrocyte-associated markers, such as RUNX2 and COL10A1, and undergo the process of endochondral ossification [6]. These findings suggest that BMSCs may not be suitable for hyaline cartilage regeneration.

Induced pluripotent stem cells (iPSCs), reprogrammed from somatic cells through the overexpression of Yamanaka factors, possess similar capabilities of self-renewal and lineage cell differentiation as embryonic stem cells [7]. iPSCs serve as an ex vivo cell source without supply limitations and can be differentiated into any cell type, including chondrocytes, for therapeutic purposes without ethical concerns. One major advantage of iPSCs over adult tissue-derived chondrocytes is their extensive expansion potential without noticeable cellular senescence. Our recent findings, demonstrating that iPSC-derived mesenchymal stem/stromal cells (iMSCs) exhibit greater chondrogenic differentiation capacity and cell proliferation with attenuated p53/p21^CIP1^ activity compared to parental synovial fluid-derived MSCs [8], indicate that iPSCs are a cell source with great regenerative potential. Particularly, iMSCs hold promise for generating naive neocartilage without a tendency for ossification [9, 10]. For example, Diederichs et al. compared the differences in chondrogenesis among iMSCs, BMSCs, and articular chondrocytes (ACs) and found that iMSC-derived chondrocytes did not undergo hypertrophic differentiation and were able to produce twofold more proteoglycans than BMSC-derived chondrocytes and control ACs [11]. These strengths have positioned iPSCs as a promising source for chondrocyte derivation in articular cartilage regeneration.

Developing autologous iPSC-based therapies has been viewed as an appealing strategy due to safety concerns. Although the allogeneic strategy using “off-the-shelf” cells or tissues is more practical for preparing grafts for implantation, concerns remain regarding the immune response against implanted cells or tissues. Studies have shown that allogeneic cartilage was infiltrated after transplantation in mice [12] and implanted allogeneic chondrocytes in a rabbit joint defect were destroyed due to a humoral immune response [13]. These results indicate that allogeneic implanted chondrocytes were attacked by host T cells and natural killer cells detecting the expression of class II major histocompatibility complex molecules and natural cytotoxicity receptors, respectively, on the chondrocytes [14, 15]. Considering the safety of cell therapies, autologous stem cells should be a more viable source than allogeneic stem cells for cartilage regeneration.

The potential of iPSC derivatives, including MSCs and chondrocytes, for articular cartilage regeneration has been explored in small mammal models, such as rodents and rabbits [16–18]. Although small animal models offer advantages in terms of cost, size, life cycle, and ease of maintenance over large animal models, the spontaneous cartilage repair observed in small animals due to their superior intrinsic healing ability may not be reproducible in studies involving large animals or clinical trials [19, 20]. On the other hand, large animal models, such as miniature pigs (minipigs), exhibit similarities in size, thickness, and anatomical structure of articular cartilage to humans [21]. Therefore, minipigs are considered a desirable animal model for translational research on cartilage regeneration.

While allogeneic and xenogeneic iPSCs have demonstrated the ability to generate cartilage in vitro and in vivo [22, 23], it remains unclear whether it would be advantageous, in terms of clinical relevance, to use autologous iPSCs for deriving therapeutic chondrocytes for articular cartilage repair in a translational animal model. In this study, we evaluated the implantation of autologous iMSC-derived chondrocytes (iMSC-Chs) and gold-standard autologous BMSC-derived chondrocytes (BMSC-Chs) for cartilage repair of the femoral condyles in skeletally matured minipigs.

## Materials and methods

**Fig. 1:**
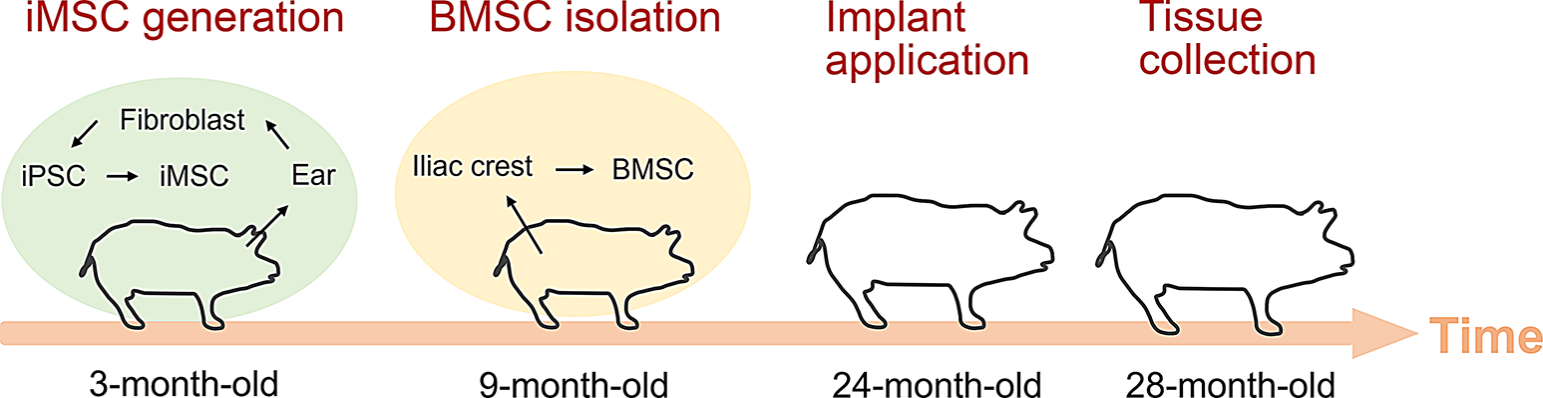
Timelines of major experimental stages for cartilage repair in minipigs. Minipig iPSCs were generated from the skin fibroblasts of 3-month-old minipigs, and BMSCs were isolated from the iliac crest of 9-month-old minipigs. All animals were maintained until the age of 2 years before undergoing autologous engineered cartilage transplantation. Four months after the transplantation, all groups, including the acellular and microfracture groups (not depicted), were euthanized to collect tissues for analysis

The in vivo study was approved by the University of Wisconsin-Madison Institutional Animal Care and Use Committee. This multi-year study was divided into several stages, starting with the generation of iMSCs and the isolation of BMSCs from juvenile minipigs for the creation of engineered cartilage implants. The process also included creating chondral defects and implanting the engineered cartilage for autologous joint repair in skeletally mature minipigs, as well as gathering tissue samples for subsequent analysis. For a comprehensive view of the experimental setup, Fig. 1 graphically represents these activities along with their corresponding timelines.

### Generation of minipig fibroblast-derived iPSCs and iMSCs

To generate autologous iPSCs, fibroblasts for cellular reprogramming were isolated from ear notch samples taken from 6 Yucatan minipigs at the age of 3 months, following our previously published protocol [24]. Specifically, the collected ear skin was digested in buffer medium supplemented with collagenase/dispase (MillporeSigma, Burlington, MA) for 2 hours. After digestion, growth culture medium composed of low-glucose Dulbecco’s Modified Eagle Medium (DMEM; Thermo Fisher Scientific, Waltham, MA, USA), 10% fetal bovine serum (FBS; Atlanta Biologicals, Atlanta, GA, USA), and 1% antibiotics (Thermo Fisher Scientific) was added to terminate the digestion process. The solution was then filtered through a 70-µm Falcon Cell Strainer (Corning, Glendale, AZ, USA) to remove undigested tissue, and cells were collected by centrifugation at 300 x g for 5 min. The cells were resuspended in growth culture medium and plated in a T75 culture flask, and then maintained in a humidified atmosphere of 5% CO2 and 95% air at 37 °C. When the cells reached 70–80% confluence, they were trypsinized using 0.05% trypsin/EDTA (Thermo Fisher Scientific) and replated at a density of 1000 cells/cm^2^. The growth culture medium was changed every 3 days.

To reprogram minipig fibroblasts into iPSCs, we transfected 6 individually isolated fibroblast lines with 4 µg of episomal plasmid (#58527; Addgene, Watertown, MA, USA) using the Nucleofector™ II with the A-024 program (Amaxa, Walkersville, MD, USA). The transfected cells were then seeded into a 6-well plate containing an irradiated mouse embryonic fibroblast (MEF; WiCell, Madison, WI, USA) feeder layer, with modified E8 medium (E8 medium (Thermo Fisher Scientific) supplemented with 5 ng/ml activin A (Thermo Fisher Scientific), 1.5 µM CHIR99021 (STEMCELL Technologies, Vancouver, BC, Canada), 2.5 µM IWR-1 (STEMCELL Technologies), and 10 ng/ml LIF (Thermo Fisher Scientific), for 21 days to induce iPSC colonies. From Day 2 to Day 7, G418 (400 µg/ml) was added to the modified E8 medium to enhance reprogramming efficacy. Under a microscope, at least two iPSC colonies per animal were hand-picked using a 27G needle and individually transferred to fresh 24-well plates. These plates contained an MEF feeder layer and modified E8 medium, which were used for the expansion and development of iPSC lines. The iPSC lines were characterized using Fast Blue RR Salt (Sigma Aldrich, St. Louis, MO, USA) staining to detect alkaline phosphatase and flow cytometry to determine the expression of pluripotency markers. Furthermore, iPSCs were differentiated into ectodermal, endodermal, and mesodermal lineage cells in vitro using the STEMdiff™ Trilineage Differentiation kit (STEMCELL Technologies) according to the manufacturer’s instructions. Subsequently, qPCR was performed to analyze the mRNA expression of representative iPSC markers and germ layer lineage-specific identifiers using the primers listed in Table 1.

**Table 1:** Minipig primer sequences for quantitative PCR analysis

Gene	Forward (5’-3’)	Reverse (5’-3’)
*UBC*	GGAGTTTGCCTGCGTTTCT	TCCAGGGTGATGGTCTTACC
*LIN28*	CCAAGGGAGACAGGTGCTAC	TGGCAGGGCTATGGATCTCT
*OCT4*	GCTGACAACAACGAGAATCTGC	ACGCGGACCACATCCTTCTCTAG
*NANOG*	AGCCCCAGCTCCAGTTTCAGC	AATGATCGTCACATATCTTCAGGCTGTA
*SOX2*	GTTCCATGGGCTCAGTGGTCAAG	AAGCGTACCGGGTTTTTCTCCATAC
*KLF4*	CCATGGGCCAAACTACCCAC	TGGGGTCAACACCATTCCGT
*PAX6*	GGTGAGAAGTGTGGGAACCG	GGCTCGAATATAGGGCTCTGA
*T*	GCTGTGAGAGGTATCCTGCC	AGCGTAAGTTGGAGAACTGCT
*GATA4*	CTAAGCAGGACCCTTGGAAC	CCATGCAGGAATCTGAGGAG
*SOX1*	CCCTCCTTCTGGTCTATTTCTC	GCAGTTTGCATCCCAGTTC
*CXCR4*	ATGGACGGGTTCCGTATATTCA	CCCGGAAACAGGGTTCCTTT
*SOX7*	GTTCATGCAGGTGAGGAGAG	GGTGGGAAGACGCTGAATAG
*PPARG*	AACATTTCACAAGAGGTGACCA	CAGCTCTCGGGAATGGGATG
*LPL*	TGACAAAGATGCCCAGTGCT	TTTCCCAAAGGACAGAATGC
*SOX9*	GACAGGAACCTGGACTTGAAAC	GTCCAAACAGGCAGAGAGATTG
*ACAN*	CTGTCTCCCTCGTGGATACTAC	CTGACACCAATGGTTCCTCTTC
*ALP*	TCCCACCACCTCCATCATAC	CAATGGCCAGCACTAAGAAGAG
*OC*	TCACACTGCTTGCCCTACT	CCTGCACCTTTGCCAGAATC
*COL2A1*	CAAGAAAGCCCTGCTCATCC	GCAGCCATCCTTCAGAACAG
*COL1A1*	GGGCAAGACAGTGATCGAATA	GGAGTTTACAGGAAGCAGACA
*COL3A1*	CTGGAGGATGGTTGCACTAAA	CCAATGTCCGCACCAAATTC
*COL10A1*	AACCAGGGAGTAACAGGAATG	CGTGAATGTGGTAGGAGAAGTAA
*RUNX2*	CTCTCTGCCAAGTGCCTATTT	ATCAGGATCGTCTCTGTCTCTC
*MMP13*	TGATGATGAAACCTGGACAAGTA	ATCTCCTGGACCATAGAGAGAC

Each of the six animal-specific iPSC lines was independently differentiated into iMSCs using the STEMdiff™ Mesenchymal Progenitor kit (STEMCELL Technologies), following the manufacturer’s instructions. Briefly, iPSCs were cultured in 24-well plates containing an MEF feeder layer with E8 medium until they reached 70–80% confluence. The culture medium was switched to STEMdiff™-ACF Mesenchymal Induction Medium (STEMCELL Technologies) for 4 days to induce iMSCs before the medium was changed to Complete MesenCult™-ACF Plus Medium (STEMCELL Technologies) for additional 2 days and then the cells were transferred into a new 24-well plate without an MEF feeder layer. The cells underwent another 4 passages using the Complete MesenCult™-ACF Plus Medium. Finally, the culture medium was switched to low glucose DMEM growth medium containing 10% FBS and 1% antibiotics to finalize the induction process. Media changes were performed daily throughout the period. All iMSC clones derived from iPSCs were collected from the induction culture without selection for specific clones with chondrogenic propensity and then expanded for this study. iMSCs from passages 5 to 7 were used, consistent with the passage numbers of the BMSCs.

### Isolation of minipig BMSCs

To harvest bone marrow from the iliac crest for BMSC isolation, 5 Yucatan minipigs at the age of 9 months were anesthetized using a combination of Telazol (5 mg/kg), ketamine (5 mg/kg), and glycopyrrolate (0.01 mg/kg) administered intramuscularly (IM). Propofol was given intravenously to the animals for intubation, followed by 2–3% isoflurane for the maintenance of general anesthesia. Preoperative doses of the antibiotic procaine penicillin G (40,000 IU/kg) and the pain medication buprenorphine (0.02 mg/kg) were administered IM. The minipigs were then transferred to a surgical suite and positioned in lateral recumbency. A 5-cm skin incision was made over a randomly selected iliac crest to expose the iliac bone using periosteal elevators. A 2 mm diameter hole was drilled on the dorsal portion of the iliac wing. Using a heparin (100 IU/ml) coated 10-ml syringe attached to a 2-mm diameter biopsy needle, 2–3 ml of bone marrow fluid was collected from the iliac bone marrow cavity. The layers of muscle, fascia, and sub-skin were closed with an absorbable suture (#4 Polysorb), and the skin was closed with a nonabsorbable suture (#3 Surgipro). A second dose of procaine penicillin G (40,000 IU/kg) was administered immediately postoperatively, prior to recovery. The isolated bone marrow fluid was transferred into a T75 flask and cultured with low glucose DMEM growth medium containing 10% FBS and 1% antibiotics, and maintained at 37 °C in a humidified atmosphere of 5% CO2. In this study, BMSCs from passages 5 to 7 were utilized.

### Cell proliferation

Proliferation of iMSCs and BMSCs seeded with or without fibrin glue/nanofiber constructs was assessed on Days 1, 4, and 7 in culture. DNA extraction from the cells was performed using Proteinase K (Millipore Sigma, Burlington, MA, USA), and the DNA content was quantified using the PicoGreen assay (Thermo Fisher Scientific), following the manufacturer’s instructions. Cumulative population doubling analysis was performed to evaluate the growth rate of long-term cell culture. The iMSCs and BMSCs were cultured and passaged when they reached 70–80% confluency for a duration of up to 100 days. The cell number at each passage was recorded and calculated. The cumulative number of population doublings was obtained by adding the calculated number from each passage to the number from the previous passage.

### Flow cytometric analysis

To characterize iPSC and MSC markers, cells were trypsinized, suspended, and washed three times using ice-cold washing buffer. The washing buffer contained 1% bovine serum albumin (Millipore Sigma), 5 mM EDTA (Thermo Fisher Scientific), and 25 mM HEPES (Thermo Fisher Scientific) in D-PBS (Cytiva, Marlborough, MA, USA). For iPSCs, the cells were incubated with primary antibodies (Thermo Fisher Scientific), including rabbit anti-OCT4, rabbit anti-NANOG, and rabbit anti-SOX2, for 30 min at 4 °C. Subsequently, the cells were washed three times with the washing buffer and then incubated with the secondary antibody (Thermo Fisher Scientific), Alexa Fluor 555 donkey anti-rabbit IgG, for an additional 30 min at 4 °C. On the other hand, iMSCs and BMSCs were incubated with Alexa Fluor 647 mouse anti-CD29 (Thermo Fisher Scientific), Alexa Fluor 488 mouse anti-CD44 (Thermo Fisher Scientific), FITC mouse anti-CD90 (BD, Franklin Lakes, NJ, USA), and PerCP-CY5.5 mouse anti-CD45 (BD) for 30 min at 4 °C. After three consecutive washes, the fluorescent cells were analyzed using Attune NxT Flow Cytometer (Thermo Fisher Scientific), and the data of marker expression were analyzed using FlowJo software (Tree Star, Ashland, OR, USA) following the manufacturer’s instructions.

### Induction of multilineage differentiation of iMSCs and BMSCs in culture

iMSCs and BMSCs were induced to differentiate into the adipogenic, chondrogenic, and osteogenic lineages for 21 days following our previously published protocol [25]. For adipogenesis, cells were plated in a 6-well plate at a density of 100,00 cells/cm² and induced by adipogenic medium consisting of high-glucose DMEM, 10% FBS, 1% antibiotics, 1 µM dexamethasone (Millipore Sigma), 0.5 mM 3-isobutyl-1-methylxanthine (Millipore Sigma), and 10 µg/ml insulin (Millipore Sigma), and 100 µM indomethacin (Sigma Aldrich). For chondrogenesis, 5 × 10⁵ iMSCs or BMSCs were collected and centrifuged at 600 x g for 5 min to form cell pellets. The pellets were then induced by chondrogenic medium consisting of high-glucose DMEM supplemented with 1% antibiotics, 1% ITS + Premix (Corning), 0.9 mM sodium pyruvate (Sigma Aldrich), L-ascorbic acid-2-phosphate (50 g/ml, Sigma Aldrich), L-proline (Sigma Aldrich), 0.1 µM dexamethasone (Sigma Aldrich), and transforming growth factor beta 1 (TGFB1) (10 ng/ml) (Thermo Fisher Scientific). For osteogenesis, cells were seeded in a 6-well plate at a density of 5 × 10³ cells/cm² and cultured with osteogenic medium containing low-glucose DMEM, 10% FBS, 1% antibiotics, 10 mM β-glycerophosphate (Sigma Aldrich), 50 µg/ml l-ascorbic acid-2-phosphate, and 0.1 µM dexamethasone. During the differentiation period, the induction medium was changed every 3 days.

After 21 days of differentiation, the cells were analyzed for mRNA expression of fat-, cartilage-, and bone-associated markers by qRT-PCR with primers listed in Table 1, histological staining, and biochemical assays. Cells induced for adipogenesis were fixed with 10% neutral buffered formalin and stained by Oil Red O (Millipore Sigma) to detect lipid droplets. The staining was imaged and then quantified by dissolving it in 100% isopropanol for the absorbance measurement at a wavelength of 515 nm to determine the lipid content. Cell pellets induced for chondrogenesis were fixed with 4% formaldehyde, dehydrated, infiltrated with xylene, and embedded in paraffin. The paraffin blocks were sectioned into 8-µm sections, deparaffinized, and stained with Alcian blue (Polysciences, Warrington, PA, USA) to detect proteoglycans. The quantification of glycosaminoglycan (GAG) was performed by digesting the cell pellets in papain solution and detected by the dimethylmethylene blue assay. The total GAG amount was normalized to the DNA content determined separately using the PicoGreen assay. For the analysis of osteogenesis, the cells were fixed with 60% isopropanol and stained by Alizarin Red S (Rowley Biochemical, Danvers, MA, USA) to detect calcium deposition. The calcium content was quantified by mixing the cells with 0.5 M hydrochloric acid (Sigma Aldrich) and measuring the mixture using the LiquiColor kit (Stanbio, Boerne, TX, USA) following the manufacturer’s instructions.

### Total RNA extraction and qRT-PCR analysis

For the analysis of mRNA expression of genes of interest, the Quick-RNA MicroPrep kit (Zymo Research, Irvine, CA, USA) was used to extract total RNA from cells. The quantity and quality of the total RNA were then measured by the Infinite M Plex system (Tecan, Männedorf, Switzerland) to prepare complementary DNA (cDNA) using the High-Capacity cDNA Reverse Transcription kit (Thermo Fisher Scientific). For real-time qPCR analysis, the cDNA samples were amplified with iQSYBR Green Premix (Bio-Rad, Hercules, CA, USA) and primers to detect specific genes of interest listed in Table 1. To determine the relative mRNA expression level of a target gene, the 2^−ΔCt^ method was employed and by which differences in cycle threshold (Ct) values between the target gene and the housekeeping *GAPDH* were calculated.

### Fabrication of cell-laden fibrin glue/nanofiber constructs

To make biomaterial scaffolds for seeing cells as implants, electrospun ultrafine nanofibers were produced following our previously published protocol [26]. Briefly, 15% (w/v) polymer solution was prepared by dissolving 90% poly-L-lactic acid (molecular weight = 50,000) (Polysciences, Warrington, PA, USA) and 10% polycaprolactone (molecular weight = 70,000–90,000) (Sigma Aldrich) in hexafluoro-2-propanol (Fluka Analytical, St. Louis, MO, USA). The solution was loaded into a 10 ml syringe fitted with a 20-gauge needle and pumped out at a speed of 1 ml/hr during electrospinning with the setup of 12-kV DC and a 15-cm electrostatic field to produce nanofibers.

For the production of three-layered sandwich constructs, a custom-made stainless-steel mold set consisting of two flat rectangular plates and one perforated 6-hole plate was employed. Each construct comprised a central component, a fibrin glue cylinder measuring 7 mm in diameter and 0.6 mm in height, surrounded by two 7-mm circular nanofibrous mats positioned above and below the fibrin glue cylinder. To create a cellular fibrin glue/nanofiber construct, iMSCs or BMSCs were trypsinized and suspended in thrombin (Baxter, Deerfield, IL, USA) at a concentration of 20 × 10^6^ cells/ml. In the mold, the bottom piece of the nanofibrous mat on a metal plate was infused with the thrombin-cell solution in a well. A combination of 5 µl of Sealer Protein Concentrate and 5 µl of the thrombin-cell solution was then added to the filled well before the top piece of the nanofibrous mat, infused with the thrombin-cell solution, was placed on top and covered with another metal plate. The assembled cellular constructs were placed in chondrogenic medium within 15-ml conical tubes for chondrogenic induction over a period of 7 days. The medium was changed every 3 days during chondrogenic differentiation. This specific timeframe was chosen for two primary reasons. Firstly, it allows the early stages of chondrogenesis to be initiated in vitro while leaving subsequent stages to occur naturally within the in vivo environment, which is more effective for complete tissue maturation. Secondly, limiting the pre-chondrogenesis phase to 7 days reduces the overall duration of in vitro culture, thereby minimizing laboratory workload and reagent utilization.

### A trocar tool designed to generate articular cartilage defects

A specialized trocar tool was custom-designed and constructed to facilitate the creation of an articular cartilage defect on the medial femoral condyle of a minipig. The trocar tool consisted of various components made from autoclavable stainless steel. To ensure stability during the procedure, a handle was incorporated into the design to securely position the trocar on the cartilage surface. The trocar tool featured an end mill, which was equipped with a stop collar and shim to regulate the depth of the defect. By employing controlled and gentle manual movements of the end mill, a precise and uniform articular cartilage defect was generated, maintaining the integrity of the underlying subchondral bone. The result was a smooth and flat surface at the base of the defect, ensuring consistency in the experimental procedure.

### Implantation of autologous iMSC- and BMSC-constructs for repair of chondral defects of minipig stifle condyles

This study adhered to ARRIVE guidelines 2.0, providing clear explanations of experimental design, minimization of bias, sample size, and statistical analyses, which aid in designing rigorous and reliable in vivo experiments. A total of nineteen 24-month-old Yucatan minipigs were randomly assigned to 4 subgroups: microfracture (*n* = 5), acellular (*n* = 3), autologous BMSC (*n* = 5), and autologous iMSC (*n* = 6). The sample size was determined to achieve a targeted statistical power of 80%. Detailed information on the animals, including identity, sex, specific ages at which different experimental procedures were performed, and weight prior to euthanasia, is provided in Table 2 for reference.

**Table 2:** Detailed information on Minipigs in Four Treatment groups

Group	ID	Sex	iPSC Generation Age (M)	BMSC Isolation Age (M)	Implantation Age (M)	Euthanasia Age (M)	Euthanasia Weight (kg)
Microfracture	9562	F	N/A	N/A	24	28	56
Microfracture	9574	F	N/A	N/A	24	28	70.8
Microfracture	9603	F	N/A	N/A	24	28	59
Microfracture	9609	F	N/A	N/A	24	28	64.8
Microfracture	9621	F	N/A	N/A	24	28	65.2
Acellular scaffold	9565	F	N/A	N/A	24	28	60
Acellular scaffold	9580	F	N/A	N/A	24	28	66
Acellular scaffold	9613	F	N/A	N/A	24	28	62.2
BMSC scaffold	742	F	N/A	9	24	28	83
BMSC scaffold	743	F	N/A	9	24	28	76
BMSC scaffold	9561	F	N/A	9	24	28	90
BMSC scaffold	9572	F	N/A	9	24	28	83
BMSC scaffold	9579	F	N/A	9	24	28	78
iMSC scaffold	9795	F	3	N/A	24	28	65.6
iMSC scaffold	9796	F	3	N/A	24	28	80.2
iMSC scaffold	9802	F	3	N/A	24	28	85.2
iMSC scaffold	9803	F	3	N/A	24	28	80
iMSC scaffold	9805	F	3	N/A	24	28	83
iMSC scaffold	9806	F	3	N/A	24	28	88.2

M: month; F: female; N/A: not applicable

A combination of Telazol (6 mg/kg) and Xylazine (2.2 mg/kg) was administered to the animals as general anesthesia prior to surgical procedures, and the animals were then given isoflurane (1–3%) and 100% oxygen at a flow rate of 2 L/min through a semi-closed circuit during surgery. The minipigs were also given Excede (5 mg/kg), a long-acting broad-spectrum antibiotic, and buprenorphine SR (0.24 mg/kg) to relieve pain. Once under anesthesia, the minipigs were positioned in dorsal recumbency and their hind limbs were suspended and prepared in a sterile manner through 3 rounds of saline solution rinsing followed by application of 4% Hibiclens. A 5-cm skin incision was made to expose a medial femoral condyle. A specially designed custom-made trocar was used to create a cylindrical cartilage-only defect measuring exactly 7 mm in diameter and 0.6 mm in depth on the weight-bearing surface of the medial femoral condyle while preserving the subchondral bone of a minipig.

For the microfracture group, 5 equally spaced 1-mm diameter holes were drilled on a cartilage defect, penetrating the subchondral bone to disrupt blood vessels to allow clot formation. For the other 3 groups, acellular, BMSC-laden, and iMSC-laden constructs were implanted at created cartilage defects and fibrin glue (Baxter) was applied to securely hold the implants in place. The layers of muscle, fascia, and sub-skin were closed using #4 − 0 Polysorb suture material, and the skin was closed using #3 − 0 Surgipro suture material. No weight-bearing reduction apparatus was used on the knee following the surgery. One month after the surgery, imaging analysis by a 3.0 T magnetic resonance imaging (MRI) scanner (GE Healthcare Discovery MR750, Waukesha, WI, USA) was performed to examine the implants within the cartilage defects. The animals were kept for a period of 4 months before being euthanized for tissue harvesting and further analysis. This time point was chosen based on existing research finding [27], which suggests it provides a balanced approach to assess both the initial outcomes of the repair and the development of any potential complications or successes.

Euthanasia was conducted in accordance with the 2020 Edition of the AVMA Guidelines for Euthanasia of Animals. For anesthetic induction, each pig received an intramuscular injection of a combination of Telazol (tiletamine HCL and zolazepam HCL) at 6 mg/kg and Xylazine at 2.2 mg/kg. Once anesthetized, an intravenous injection of the euthanasia drug was administered at a dosage of 1 ml per 4.5 kg body weight, using either Fatal-Plus (sodium pentobarbital solution) or Euthasol. Euthanasia was confirmed by verifying the cessation of respiratory and cardiac functions.

### Analysis of cytokines in joint cavities

The synovial fluid and stifle joints of minipigs were collected by a trained veterinarian for cytokine analysis and histological evaluation, respectively. After euthanizing each minipig, synovial fluid was collected under sterile conditions. Initially, the skin around the stifle joint was cleaned and sterilized. The joint was then opened using a surgical blade, and synovial fluid was drawn using an 18-gauge needle attached to a 5 ml syringe, ensuring a minimum collection of 500 µl. The collected fluid was immediately placed on ice to maintain the integrity of its components and subsequently stored at -80 °C before analysis. Levels of inflammation-associated cytokines were assessed by MILLIPLEX MAP Porcine Cytokine/Chemokine Magnetic Bead Panel – Immunology Multiplex Assay (Millipore Sigma), following the manufacturer’s instructions.

### Assessment of mechanical property of regenerated cartilage

The mechanical property of neo-cartilage generated at defects was determined by the measurement of tissue stiffness using the hand-held arthroscopic indentation probe Artscan 200 (Artscan Oy, Helsinki, Finland) in accordance with the manufacturer’s instructions. The Young’s modulus (E) of the tissue was calculated based on the equation E = F(1-ν)Rχ / (4α3κ), where F represents the force measured from the probe, ν is the Poisson’s ratio (assumed to be 0.5), R denotes the radius of curvature of the indenter (0.35 mm), α corresponds to the height of the indenter (0.13 mm), and κ represents the theoretical correction factor [28]. We used intact cartilage of the contralateral knee in the tested groups as a reference control for comparison.

### Histological assessment of repaired cartilage

Stifle joint specimens were fixed in 10% neutral-buffered formalin and decalcified in 10% neutral-buffered EDTA. After the completion of decalcification was confirmed by radiography (Faxitron UltraFocus DXA, Tucson, AZ, USA), the specimens were embedded in paraffin and sectioned into 8-µm slices for subsequent histological and immunohistochemical staining. Hematoxylin and eosin (H&E) and Safranin O/Fast Green staining were used to analyze the cellular structure and cartilage matrix of the repaired tissue across different groups. Intact cartilage harvested from the contralateral knee in the microfracture group served as a reference control for comparison. To quantitatively assess cartilage repair, optical macrographs and histological micrographs of the specimens were evaluated by 4 reviewers independently, who were blinded to the experimental groups. The ICRS-I and ICRS-II scoring systems were employed for the assessment of macrographs and micrographs, respectively. The reported scores in the results section represent the mean values calculated from assessments made by the independent reviewers.

To stain samples for immunohistochemical analysis, the sectioned slices were deparaffinized and subjected to unmasking by 0.1% (w/v) pepsin (Sigma Aldrich) in 0.01 N HCl for 15 min. Subsequently, permeabilization was performed using 0.1% Triton X-100 (Thermo Fisher Scientific) in D-PBS for 30 min, followed by blocking with ice-cold 0.1% (w/v) BSA in D-PBS for 30 min at room temperature. The specimen slides were then incubated with the primary antibodies, goat anti-human COL1A1 (Santa Cruz Biotechnology, Dallas, TX, USA), COL2A1 (Santa Cruz Biotechnology), and COL10A1 (Santa Cruz Biotechnology) at a 1:200 ratio for 1 h at 4 °C. Afterward, the slides were washed 3 times with ice-cold D-PBS to remove unbound antibodies and then incubated with the secondary antibody FITC donkey anti-goat (Santa Cruz Biotechnology) at a 1:200 ratio for 30 min at 4 °C. Following 3 additional washes with ice-cold D-PBS to remove unbound secondary antibody, the slides were mounted with coverslips using mounting medium containing DAPI (Vector, Burlingame, CA, USA). Fluorescence imaging of the specimens was conducted using a Nikon A1R-s confocal microscope (Nikon, Tokyo, Japan). The fluorescence intensity of the images, which indicates the expression levels of COL1A1, COL2A1, and COL10A1 across different groups, was quantified using an established method [29].

### Statistical analysis

All the results presented in this study were derived from a minimum of three independent biological replicates (*n* = 3). Quantified data were expressed as means ± standard deviation (SD), and statistical comparisons between groups were performed using Student’s t-test or one-way analysis of variance (ANOVA) followed by a post hoc Tukey’s test. A significance level of *p* < 0.05 was considered statistically significant and used to determine the presence of significant differences among the experimental groups.

## Results

### Pluripotent characteristics of minipig fibroblast-derived iPSC

**Fig. 2:**
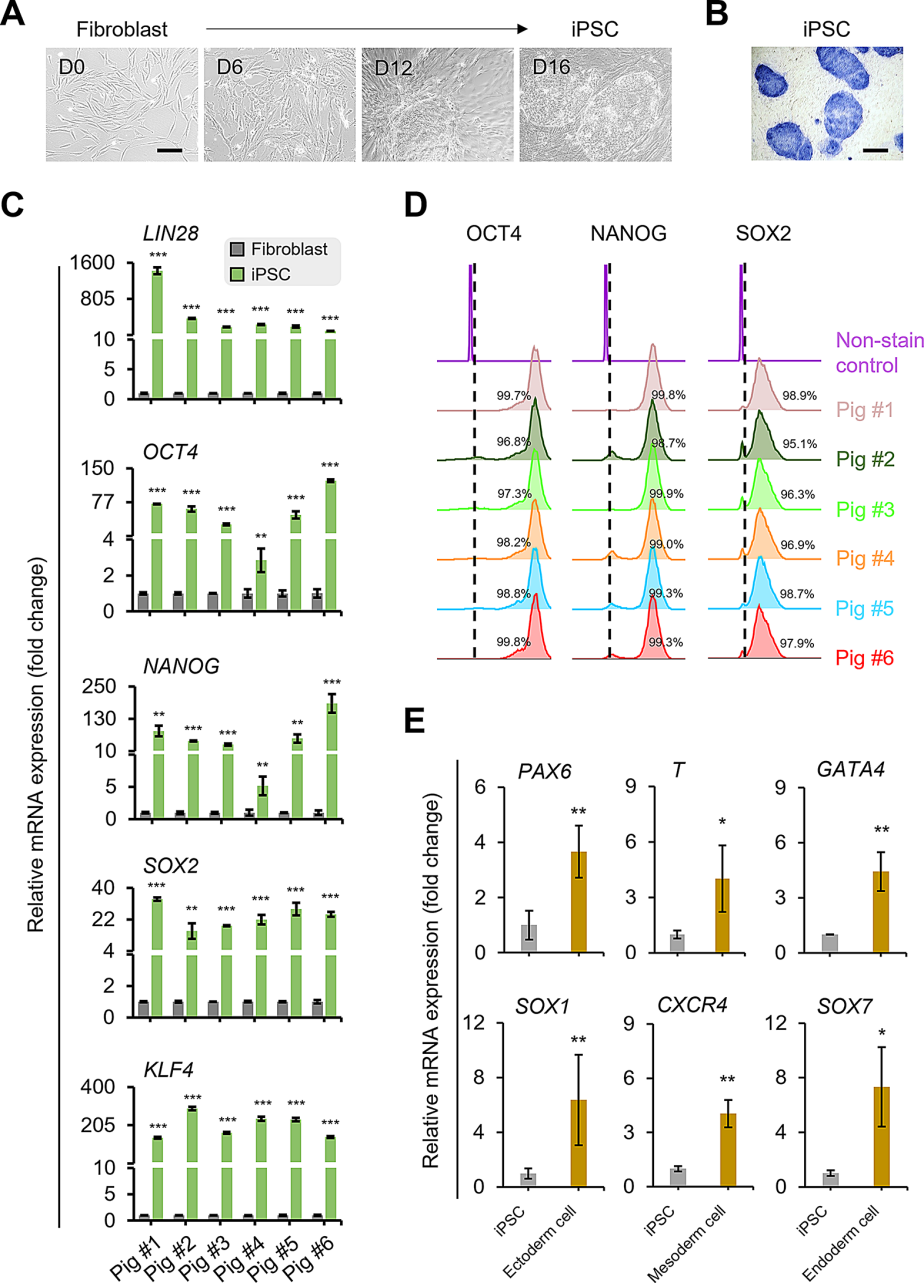
Characterization of minipig iPSCs reprogrammed from dermal fibroblasts. (**A**) Changes in cell morphology from minipig fibroblasts to iPSCs during cellular reprogramming between days 0 and 16. (**B**) Representative iPSC colonies stained with alkaline phosphatase for determination of pluripotency. (**C**) Transcript levels of pluripotency markers in fibroblasts and iPSCs individually derived from 6 minipigs. (**D**) Flow cytometry analysis of expression of pluripotency markers in 6 independent iPSC lines. (**E**) Transcript levels of ectodermal markers *PAX6* and *SOX1*, mesodermal markers *T* and *CXCR4*, and endodermal markers *GATA4* and *SOX7*. Scale bar = 200 μm. *p < 0.05, **p < 0.01, ***p < 0.001; *n* = 6 biological replicates

To generate minipig iPSCs, we isolated fibroblasts from the ears of 6 animal and transfected them with the episomal plasmid pMaster12 to ectopically overexpress pluripotency factors for cellular reprogramming. At day 1 after transfection, the fibroblasts exhibited an elongated morphology, gradually transitioning into iPSCs with epithelial morphology. By day 16, tightly packed ESC-like colonies with well-defined borders had formed (Fig. 2A). After 21 days, we hand-picked the iPSC colonies and transferred them to culture plates coated with a feeder layer of mouse embryonic fibroblasts to maintain the established cell lines.

To characterize the iPSCs, we performed staining for alkaline phosphatase (ALP). The iPSC colonies consistently showed positive ALP staining (Fig. 2B). We further examined the expression levels of pluripotency markers using qPCR and flow cytometry. The transcript expression of *LIN28*, *OCT4*, *NANOG*, *SOX2*, and *KLF4* was significantly upregulated in iPSCs compared to their parental fibroblasts (Fig. 2C). Flow cytometry analysis revealed that over 95% of the iPSCs derived from each minipig showed positive staining for the markers OCT4, NANOG, and SOX2 (Fig. 2D). In addition to exhibiting pluripotent characteristics, the iPSCs demonstrated the ability to differentiate into cells of the three germ layers. After 7 days of induction with lineage-specific differentiation medium, the minipig iPSCs expressed significantly higher levels of ectoderm-associated markers *PAX6* and *SOX1*, mesoderm-associated markers *T* and *CXCR4*, and endoderm-associated markers *GATA4* and *SOX7* compared to non-induced iPSC controls (Fig. 2E). Taken together, these results indicate that using our protocol, we successfully generated autologous minipig iPSCs from fibroblasts.

### Multilineage differentiation capability of iMSCs and BMSCs

**Fig. 3:**
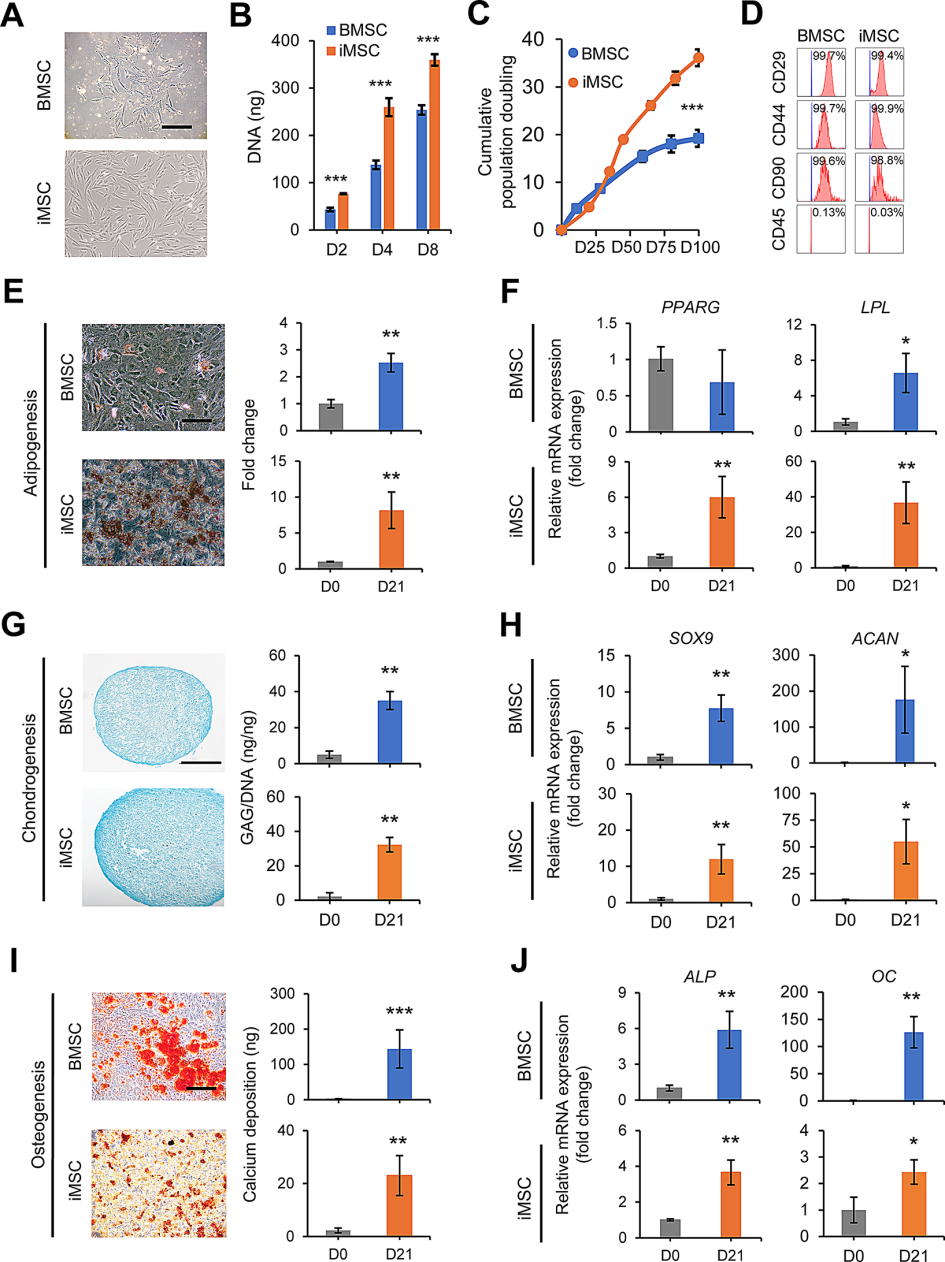
Characterization of minipig iMSCs and BMSCs. (**A**) Morphology of iMSCs and BMSCs. (**B**) Proliferation of iMSCs and BMSCs measured by DNA content at different time points. (**C**) Cumulative population doubling levels of iMSCs and BMSCs measured at each passage up to 100 days. (**D**) Flow cytometry analysis of iMSCs and BMSCs for detection of MSC-associated surface markers (CD29, CD44, and CD90) and hematopoietic marker (CD45). (**E**) Oil red O staining and quantification of lipid droplets following adipogenesis, and (**F**) transcript levels of adipose-associated markers (*PPARG* and *LPL*) in iMSCs and BMSCs after adipogenesis. (**G**) Alcian blue staining and quantification of glycosaminoglycans (GAGs) following chondrogenesis, and (**H**) transcript levels of cartilage-associated markers (*SOX9* and *ACAN*) in iMSCs and BMSCs after chondrogenesis. (**I**) Alizarin red S staining and quantification of calcium deposition following osteogenesis, and (**J**) transcript levels of bone-associated markers (*ALP* and *OC*) in iMSCs and BMSCs after osteogenesis. Scale bar = 200 μm. *p < 0.05, **p < 0.01, ***p < 0.001; *n* = 3 biological replicates

Minipig iPSCs were induced to differentiate into iMSCs using the STEMdiff™ Mesenchymal Progenitor kit, while BMSCs were isolated from bone marrow following our laboratory protocol. Morphology analysis revealed that both iMSCs and BMSCs exhibited a similar spindle shape (Fig. 3A). In terms of cell growth, iMSCs displayed a higher proliferation rate in short-term culture (8 days) (Fig. 3B) and accumulated more population doublings in long-term culture (100 days) compared to BMSCs (Fig. 3C). Both iMSCs and BMSCs expressed MSC-associated cell surface antigens, including CD29, CD44, and CD90, while lacking the hematopoietic-associated marker CD45 (Fig. 3D). To assess the differentiation potential of cells, iMSCs and BMSCs were induced for adipogenesis, chondrogenesis, and osteogenesis. Adipogenic induction resulted in the formation of lipid droplets in both iMSC and BMSC cultures, as detected by Oil red O staining and quantification (Fig. 3E). Additionally, the expression of the adipocyte-associated marker *LPL* was significantly elevated in both induced BMSCs and iMSCs, while *PPARG* expression was significantly increased only in iMSCs (Fig. 3F), indicating adipocyte differentiation in both cell types, with higher efficiency observed in iMSCs. For chondrogenesis, iMSCs and BMSCs demonstrated the ability to form chondrocytes, as evidenced by Alcian blue staining for GAG production (Fig. 3G). Quantification using the dimethylmethylene blue assay, normalized to DNA content, confirmed the presence of GAG in the induced cells. Moreover, chondrogenic induction led to increased expression of chondrocyte-associated markers *SOX9* and *ACAN* in both cell types (Fig. 3H). iMSC-Chs did not express markers associated with hypertrophic chondrocytes and fibrocartilage chondrocytes. In contrast, during the 21-day chondrogenesis, BMSC-Chs showed increased levels of *MMP13*, *RUNX2*, and *COL10A1*, indicative of chondrocyte hypertrophy (Fig. S1). In terms of osteogenesis, both iMSCs and BMSCs exhibited the differentiation into osteoblasts, as indicated by Alizarin red staining for mineral deposition and calcium quantification (Fig. 3I). Additionally, the expression of osteoblast-associated markers *ALP* and *OC* was observed in both cell types upon osteogenic induction (Fig. 3J). These results demonstrated the multilineage differentiation potential of both iMSCs and BMSCs.

**Fig. 4:**
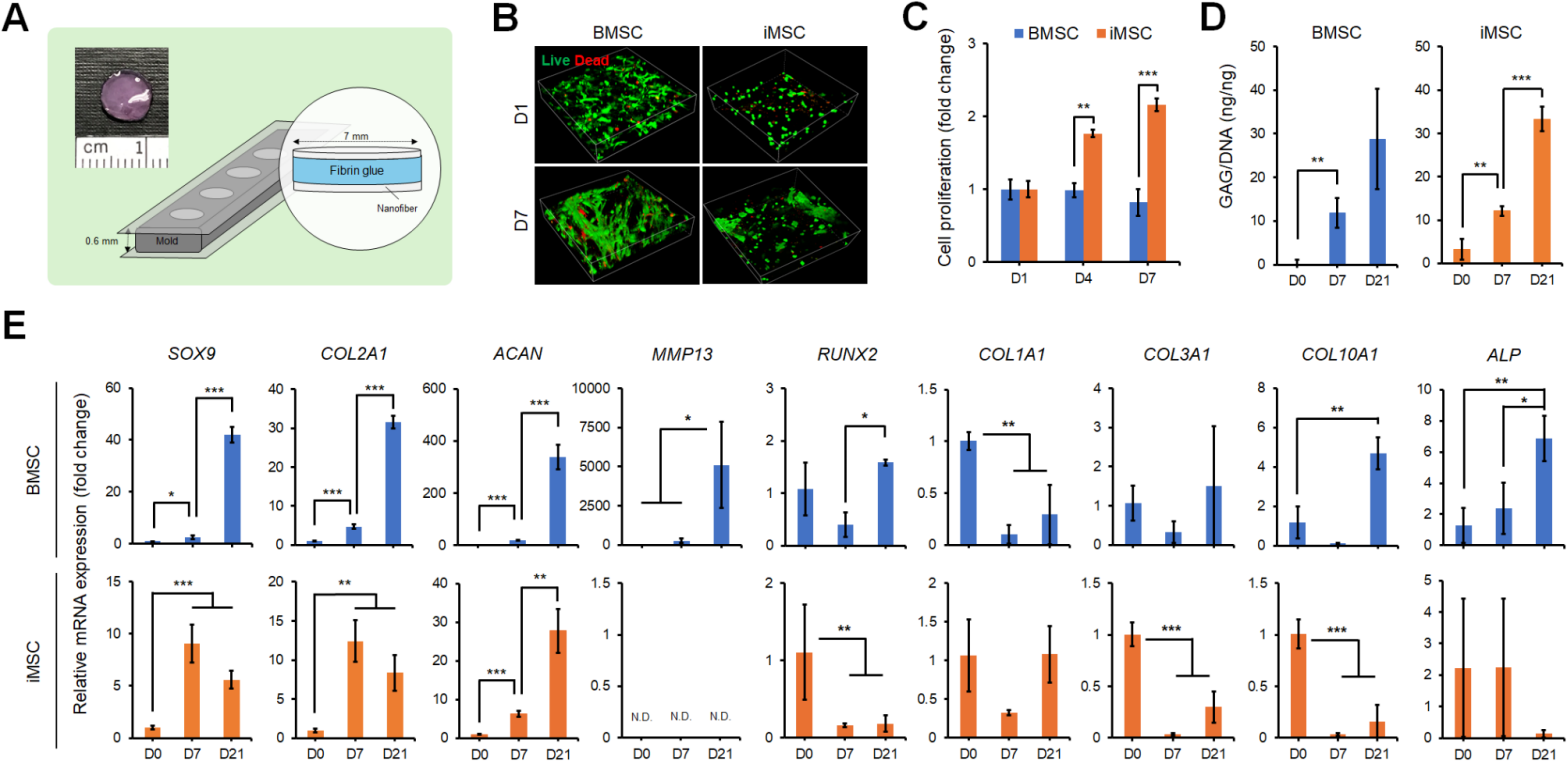
Chondrogenic evaluation of iMSCs and BMSCs seeded in fibrin glue/nanofiber constructs. (**A**) Schematic of the fibrin glue/nanofiber sandwich construct, where iMSCs or BMSCs are mixed with fibrin glue and assembled with nanofibrous mats to assess chondrogenic capacity. (**B**) Live (green) and dead (red) staining of iMSCs or BMSCs cultured in the fibrin glue/nanofiber construct at different time points. (**C**) Proliferation of iMSCs and BMSCs cultured in the construct, measured by DNA content at days 1, 4, and 7. (**D**) Quantification of GAG production per DNA content of iMSCs and BMSCs following chondrogenesis in the sandwich construct, measured at different time points. (**E**) Transcript levels of hyaline cartilage-associated markers (*SOX9*, *COL2A1*, and *ACAN*), hypertrophic chondrocyte-associated markers (*MMP13*, *RUNX2*, *COL10A1*, and *ALP*), fibrocartilage-associated marker (*COL1A1* and *COL3A1*) during chondrogenic differentiation of iMSCs or BMSCs at different time points. *p < 0.05, **p < 0.01, ***p < 0.001; *n* = 3 biological replicates. N.D.: not detected

We manufactured a biomaterial construct consisting of fibrin glue hydrogel mixed with iMSCs or BMSCs enclosed by nanofibrous mats (Fig. 4A) and induced for chondrogenesis to generate an engineered cartilage implant for chondral defect repair. Live/dead assay demonstrated high cell viability in both iMSC and BMSC constructs with live cells stained green and dead cells stained red (Fig. 4B). Proliferation analysis based on total DNA content revealed distinct growth patterns in iMSC and BMSC constructs during chondrogenic induction. iMSC constructs exhibited an increase in cell number within the initial 7 days, while BMSC constructs remained stable (Fig. 4C). After 21 days, both iMSC and BMSC constructs transformed into engineered cartilage-like implants, characterized by a significant increase in GAG content (Fig. 4D). To assess the phenotype of differentiated chondrocytes in these constructs, we analyzed the mRNA expression of markers associated with hyaline cartilage, fibrocartilage, and hypertrophic chondrocytes. The expression of key hyaline cartilage markers, including *SOX9*, *COL2A1*, and *ACAN*, was significantly upregulated in chondrocytes derived from both iMSC and BMSC constructs (Fig. 4E). In contrast, hypertrophic chondrocyte markers, such as *MMP13*, *COL10A1*, and *ALP*, were significantly upregulated in the BMSC constructs, while the iMSC constructs showed no such increase. Regarding fibrocartilage markers, *COL1A1* and *COL3A1* levels were either reduced or remained low in both cell types during the 21-day chondrogenic induction. These findings indicate the generation of hyaline cartilage chondrocyte-laden implants from iMSC constructs and hypertrophic chondrocyte-laden implants from BMSC constructs in our in vitro model.

### Secure retention of engineered cartilage implants in chondral defects of minipig femoral condyles

**Fig. 5:**
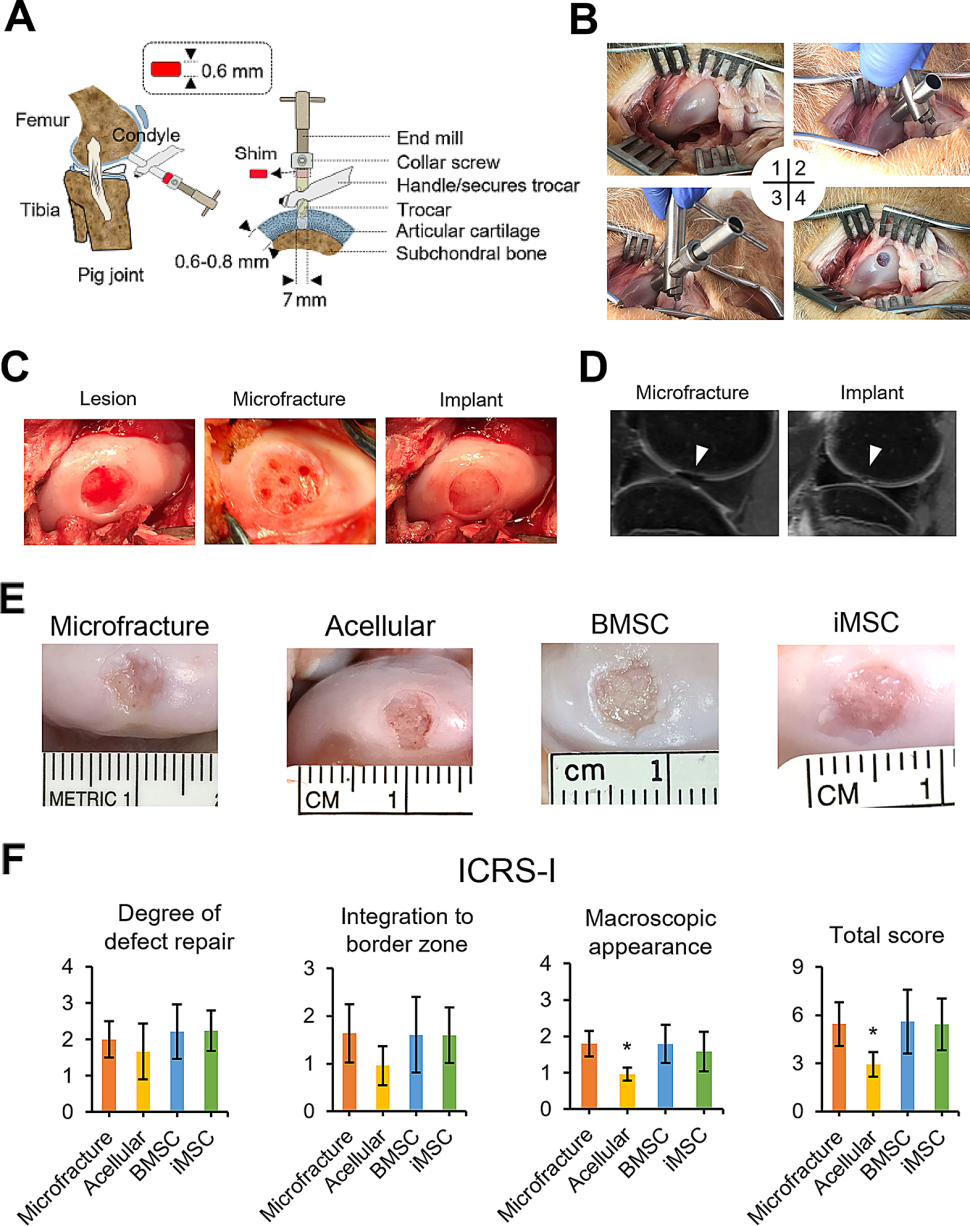
Chondral defects on medial femoral condyles, engineered cartilage construct implantation, and macroscopic assessment of articular cartilage repair. (**A**) Illustration of the custom-designed trocar tool utilized to create a critical-sized articular cartilage defect, measuring 7 mm in diameter and 0.6 mm in depth, while preserving the integrity of the subchondral bone. (**B**) Step-by-step procedure for creating an articular cartilage defect in a minipig as follows: (1) A 10-cm skin incision was made to expose the defect site on the weight-bearing surface of the medial femoral condyle. (2) The trocar tool was placed on the surface of the articular cartilage. (3) The end mill was inserted into the trocar along with a 0.6 mm thick shim and a secure collar. (4) The shim was then removed, and gentle back and forth movements of the end mill were applied to create a critical-sized articular cartilage defect with a diameter of 7 mm and a depth of 0.6 mm. (**C**) Representative images of chondral lesions generated by the trocar tool prior to scaffold implantation or microfracture drilling (all groups; left), after microfracture drilling (microfracture group; middle), and after engineered construct implantation (acellular and cellular groups; right). (**D**) MRI images captured 1 month after surgery demonstrating the repaired status of the defects in the microfracture and construct implantation groups. (**E**) Representative macroscopic images of the repaired articular cartilage lesions in minipigs, taken 4 months after the surgical procedure. The images portray the repair outcomes of different treatment groups, including the microfracture group (clinical treatment control) and the acellular group (cellular treatment control). (**F**) The ICRS-I scoring system utilized for the visual assessment of the repaired articular cartilage in minipigs. The graph presents the scores obtained from the evaluation, with statistical significance indicated by asterisks (*p < 0.05). The data reflects a sample size of 5 biological replicates

To create a standardized chondral defect suitable for implanting engineered cartilage, we employed a specially designed hand-held trocar tool (Fig. 5A). This tool allowed us to create a circular lesion with a consistent depth, measuring 7 mm in diameter and 0.6 mm in depth, specifically on the weight-bearing surface of the medial femoral condyle. Importantly, we ensured that the subchondral bone remained intact and unaffected (Fig. 5B). Following the creation of the chondral defect, we introduced the engineered cartilage implant derived from either a BMSC- or iMSC-laden construct, or an acellular fibrin glue/nanofibrous mat construct. Prior to implantation, the cartilage implant was coated with fibrin glue to enhance its stability within the defect. For comparison, we included a control group that underwent the well-established microfracture technique, producing evenly spaced subchondral holes with a diameter of 1 mm (Fig. 5C). Postoperative MRI images taken after one month confirmed the secure placement of the implants within the targeted cartilage defect sites in the minipig femoral condyles (Fig. 5D).

Four months after the implantation surgery, we conducted a macroscopic evaluation of critical-sized chondral defects repaired by engineered cartilage. Macroscopic analysis of the harvested joints revealed partial filling of the cartilage defects in the cell-laden construct and microfracture groups, whereas the acellular construct group showed little to none neo-tissue generated in the defects (Fig. 5E). Quantitatively, the acellular group received significantly lower scores based on visual assessment using the ICRS-I scoring system compared to the other three groups (Fig. 5F).

### iMSC-Ch implants facilitate regeneration of hyaline cartilage in chondral defects of femoral condyles

**Fig. 6:**
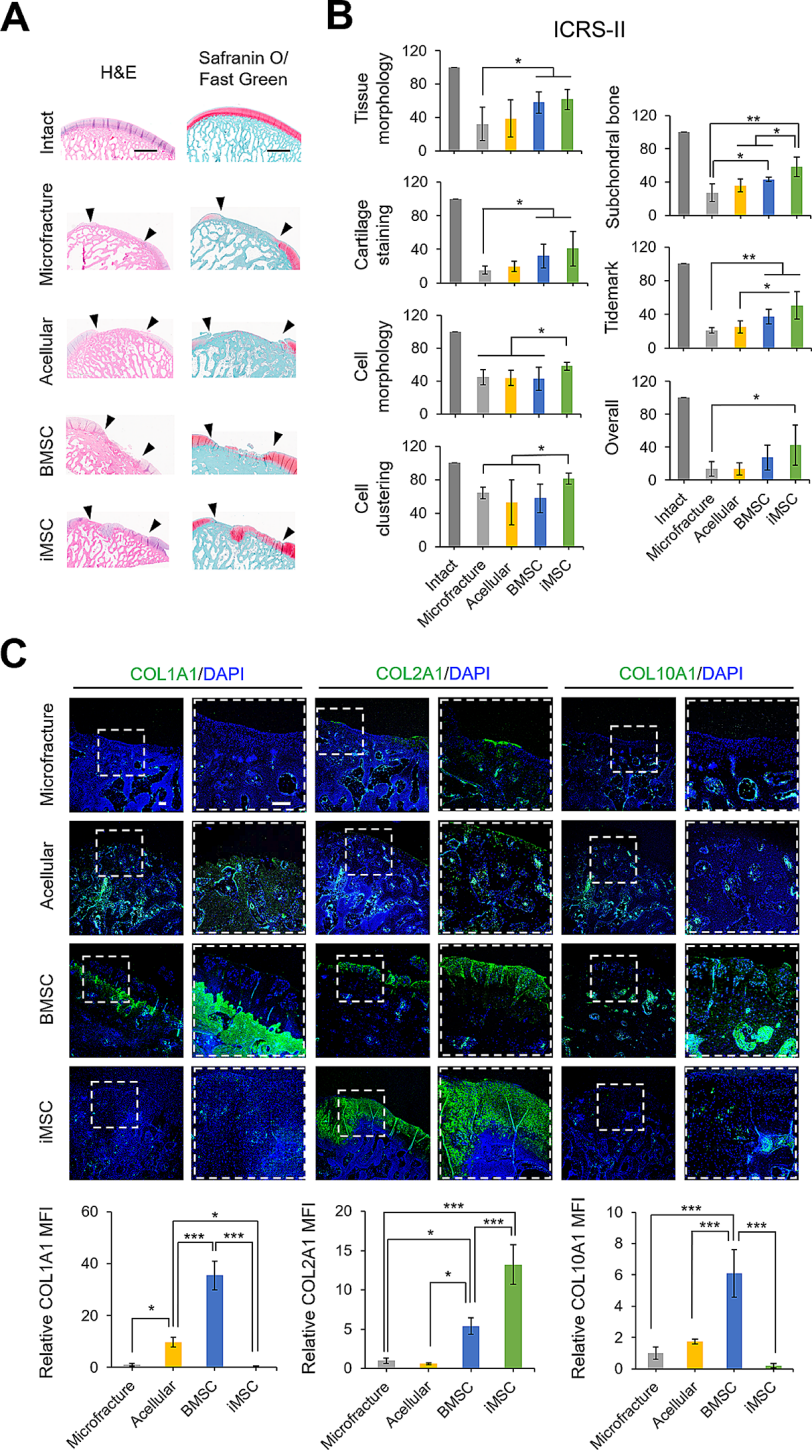
Histological assessment of articular cartilage repair. (**A**) H&E-stained images showing the histological assessment of the articular cartilage defect in minipigs, taken 4 months after surgery. Safranin O/Fast Green staining performed to analyze cartilage regeneration, with cartilage matrix stained red and other connective tissues stained green. Intact cartilage serves as the reference control, and the site of the created defect is indicated by black arrows. (**B**) The histological assessment of the regenerated minipig articular cartilage conducted using the ICRS-II grading score. The graph presents the scores obtained from the evaluation, with statistical significance indicated by asterisks. The assessment was performed on a sample size of *n* = 5. (**C**) Immunofluorescence staining performed on the regenerated minipig articular cartilage to detect the presence of cartilage-associated markers, including COL1A1, COL2A1, and COL10A1. The magnified images on the right represent regions enclosed by dashed boxes in the left columns. The relative mean fluorescence intensity (MFI) was used to quantify the immunofluorescence staining of the markers. The nuclear DNA is labeled with DAPI. The scale bar in the images corresponds to a length of 200 μm. *p < 0.05, **p < 0.01, ***p < 0.001; *n* = 5 biological replicates

Histological analysis with H&E staining showed new tissue generation partially covering the chondral defects in the BMSC-Ch, iMSC-Ch, and microfracture groups, with minimal tissue repair in the acellular group, in reference to intact cartilage (Fig. 6A). Alterations of the subchondral bone structure, likely caused by changes in the mechanical environment of a repaired joint, were found in some animals of each group. In terms of neocartilage matrix formation, Safranin O/Fast Green staining demonstrated more regenerated cartilage in the iMSC-Ch group compared to that in the BMSC-Ch group, whereas the microfracture and acellular groups showed little or no cartilage regeneration (Fig. 6A). Quantitative histological assessment using the ICRS-II scoring system revealed that both the BMSC-Ch and iMSC-Ch groups achieved significantly higher scores in the parameters of tissue morphology, cartilage staining, subchondral bone, and tidemark compared to the microfracture control group (Fig. 6B). When comparing the ICRS-II scores between the two cellular implant groups, the repaired cartilage by iMSC-Ch implants received higher scores in the parameters of cell morphology, cell clustering, and subchondral bone regeneration compared to that by BMSC-Ch implants.

Immunofluorescence staining of cartilage-associated markers demonstrated that while there was a lack of regenerated cartilage matrix in the microfracture and acellular groups, the cellular implant groups showed the production of collagenous matrix (Fig. 6C). Quantitative analysis revealed that regenerated cartilage from iMSC-Ch implants contained significantly higher levels of COL2A1, with almost undetectable levels of COL1A1 and COL10A1, compared to the other three treatments. In contrast, tissue repair from BMSC-Ch implants resulted in tissue rich in COL1A1, COL2A1, and COL10A1. Taken together, our findings demonstrate that the cellular implant groups showed enhanced cartilage repair compared to the microfracture or acellular groups. iMSC-Ch implants specifically promoted the regeneration of hyaline cartilage-like tissue, whereas BMSC-Ch implants resulted in the formation of fibrocartilage and cartilage containing hypertrophic chondrocytes.

### Reduction of inflammation in joints repaired by iMSC-Ch implants

**Fig. 7:**
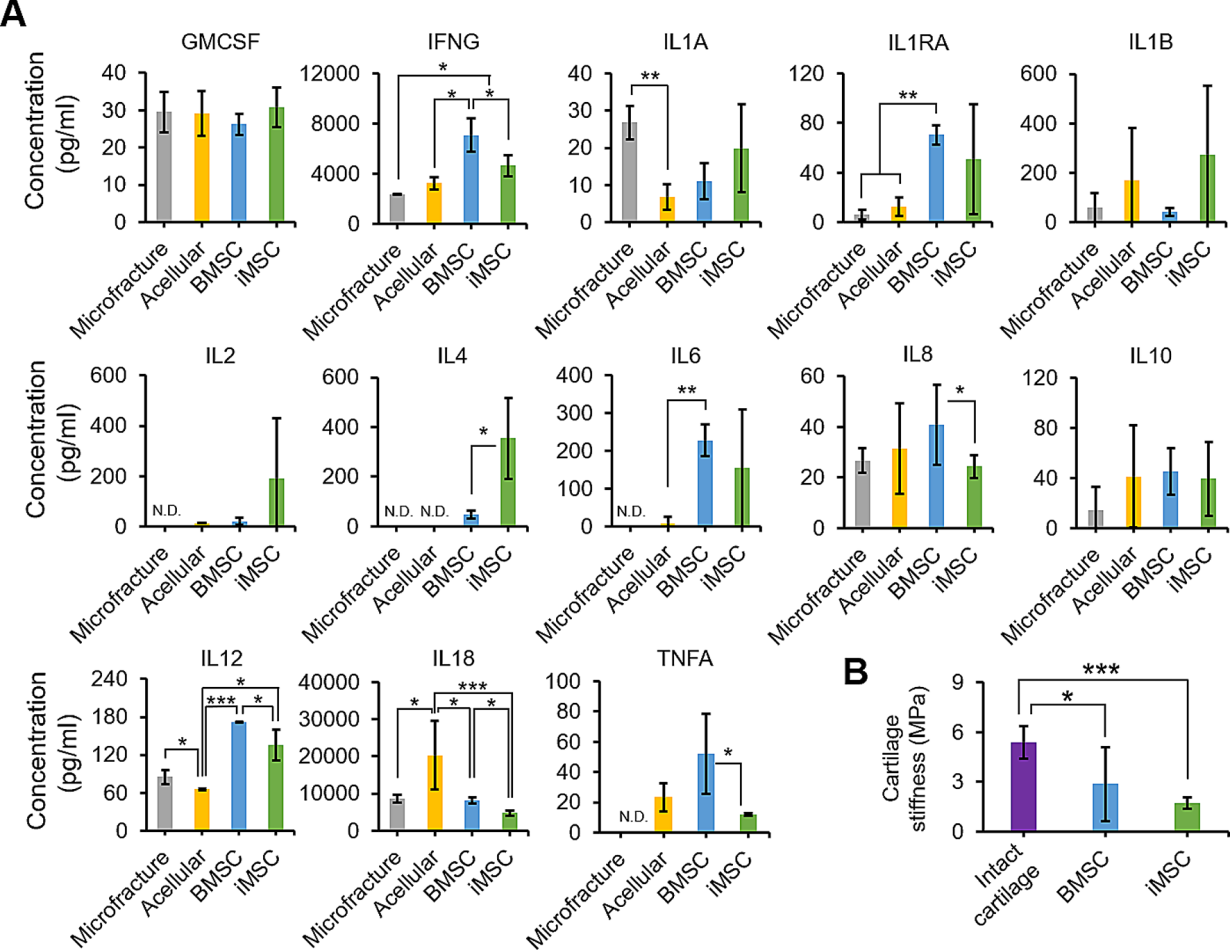
Inflammatory cytokine levels in synovial fluid and stiffness of regenerated cartilage in repaired joints. (**A**) Assessment of anti- and pro-inflammatory marker expression in synovial fluid from repaired joints of minipigs. The expression levels of these markers were evaluated to analyze the microenvironment within the repaired joints. (**B**) The indentation hardness of both intact and repaired cartilage measured to assess their mechanical characteristics. Statistical significance is indicated by asterisks. *p < 0.05, **p < 0.01, ***p < 0.001; *n* = 5 biological replicates. N.D.: not detected

We analyzed inflammation-associated cytokines in the joint microenvironment of minipigs treated with microfracture, acellular implants, or cellular implants. The results revealed a complex interaction of pro- and anti-inflammatory molecules during cartilage repair across the different treatment groups, as indicated by mixed trends in cytokine levels (Fig. 7A). Specifically, there was no statistically significant difference in the levels of GMCSF, IL1B, IL2, and IL10 across all four groups. Cytokine levels in the implant groups were generally higher than those in the microfracture group, except for IL1A, likely due to the pro-inflammatory effect of fibrin glue/nanofibers. Comparing the acellular and cellular implant groups, the cellular implants showed increased levels of anti-inflammatory factors (IL1RA, IL4, and IL6) and reduced levels of the pro-inflammatory cytokine IL18, suggesting a potential immunomodulatory effect from BMSCs or iMSCs. The significant elevation of IFNG and IL12 in the cellular implant groups compared to the acellular group remains unclear. Furthermore, when comparing the cellular implant groups, minipigs receiving iMSC-Ch implants showed lower levels of pro-inflammatory factors (IFNG, IL8, IL12, IL18, and TNFA) and higher levels of the anti-inflammatory factor IL4 compared to those receiving BMSC-Ch implants, indicating potentially enhanced immunomodulatory effects of iMSC-Chs.

In order to assess the mechanical properties of the repaired cartilage, we measured the stiffness of both regenerated and intact cartilage as a reference control (Fig. 7B). Our results indicated that the stiffness of regenerated articular cartilage derived from iMSC-Ch implants was comparable to that of BMSC-Ch implants. However, it is worth noting that the regenerated cartilage exhibited significantly lower stiffness compared to intact cartilage. These findings suggest that iMSC-Ch implants possess the ability to modulate the inflammatory response in a repaired joint, although further enhancements in their mechanical properties are necessary.

## Discussion

The significance of this study is to evaluate the feasibility of using ex vivo-generated autologous iPSC-derived chondrocytes for repairing critical-sized chondral defects in a large animal model. To the best of our knowledge, this study is the first to address the question of whether the autologous iPSC strategy is viable for cartilage repair. We generated transgene-free minipig iPSCs using the non-integrating episomal approach [24] to demonstrate their potential for clinical applications. Additionally, we included groups of BMSCs and microfracture, representing the gold standard cell type and surgical intervention, respectively, as controls to compare the outcome of cartilage repair by iPSC derivatives. Our major findings revealed that iPSC-derived chondrocytes exhibit higher proliferation rates than BMSC-derived chondrocytes, while both types show a similar capacity for cartilaginous matrix production. Particularly, autologous iMSC-laden implants promote hyaline cartilage formation and enhance the immune microenvironment of the joint compared to autologous BMSC-laden implants. These findings demonstrate the potential of autologous iPSCs as a source of therapeutic cells for articular cartilage repair.

The optimal choice between autologous and allogeneic cell sources for cartilage repair therapies remains a topic of ongoing investigation due to the distinct advantages and limitations associated with each approach. Autologous cell transplantation is considered a safer option compared to allogeneic transplantation. However, the high costs involved in generating patient-specific cells meeting cGMP requirements restrict the clinical feasibility of autologous approaches [30]. On the other hand, allogeneic “off-the-shelf” graft implantation provides a more cost-effective alternative with readily available cell products and an abundant supply of cells. Nevertheless, the risk of immune rejection presents a significant challenge to successful allogeneic cell transplantation [31, 32], even with human leukocyte antigen (HLA) matching, necessitating the use of immunosuppressive medications that carry increased infection and cancer risks [33]. Excitingly, a recent study conducted by Tsumaki’s group has demonstrated promising results in a primate animal model using allogenic iPSC-derived cartilage implants [34]. These implants successfully filled chondral defects and transformed into white articular cartilage, resembling the surrounding tissue. These findings help address safety concerns associated with the use of allogenic iPSCs for cartilage reconstruction. However, it is essential to note that the previous study primarily focused on relatively small cartilage lesions measuring 1 mm in diameter and 0.5 mm in depth. Further investigation is necessary to determine if similar repair outcomes can be achieved in a large animal model with critical-sized defects, as employed in our study. Studying critical-sized defects is crucial as they present greater challenges in terms of mechanical load distribution and inflammation associated with the lesion, which necessitate achieving satisfactory repair outcomes. An emerging trend in this research field is the application of CRISPR/Cas9 gene-editing technology to disrupt human HLA genes, thereby creating immunocompatible iPSCs capable of overcoming immune rejection challenges during allogeneic cell transplantation [35, 36]. This development is expected to mitigate the safety concerns associated with the immunogenicity of allogeneic iPSCs, while still harnessing the advantages of off-the-shelf cell therapy for cartilage repair.

In this study, we implemented autologous cell implants and transgene-free cellular reprogramming to address safety concerns associated with immunogenicity and tumorigenicity in iPSC-derived cell therapies [37, 38]. Our findings indicate that minipigs receiving cartilage implants generated from their own BMSC- or iPSC-derived chondrocytes experienced minimal immune response-related inflammation and pain. Moreover, no tumor formation was observed at the defect site after iMSC implantation. The utilization of an integration-free reprogramming method likely contributed to this positive outcome. A report suggests that integrating methods using recombinant DNA or viral vectors can lead to genomic disruptions and oncogenic activations [39]. In contrast, non-integrating methods like episomal plasmids, although having lower transfection efficiencies, are considered reliable and safe for translational research and future clinical applications. It is important to note that the pMaster12 plasmid utilized in this study for iPSC reprogramming contains the MYC transcription factor. MYC can enhance cell reprogramming efficiency by regulating cell metabolism during early reprogramming stages through the induction of a robust hybrid energetics program [40], although it has been shown to be associated with the promotion of undesired tumor formation. Our study confirmed that all iMSC lines were transgene-free, indicating the absence of the four exogenous Yamanaka factors. This outcome alleviates safety concerns regarding the presence of these factors in the implanted cells.

The innovation of our study lies in using a large animal model to demonstrate both the procedure and outcomes of autologous iPSC therapy for cartilage repair, thereby serving as translational research that paves the way for clinical applications. In parallel, another research team has explored the use of xenograft human iPSC-derived cells to regenerate articular cartilage in 6-month-old minipigs [41]. While the use of xenografts to evaluate the potential of human cells in cartilage regeneration is promising, the interaction between the pig joint microenvironment and human cells requires further investigation. Additionally, the xenograft approach to cartilage repair demands a more comprehensive assessment to determine its clinical relevance. In contrast, our approach of autologous cell transplantation offers valuable insights into a model of autologous iPSC-based cartilage regeneration that could inform future clinical trials. Particularly, our findings suggest a potential trend where implanted iPSC-derived cells may reduce inflammation and promote anti-inflammatory activities, though further studies are needed to directly assess and confirm their enhanced immunomodulatory effects. This aligns with previous research, where the implantation of iPSC-MSC-derived chondrocytes significantly reduced inflammatory markers, such as IL1B and TNFA, in an osteoarthritis rabbit model [42]. Together, these findings underscore the potential of iPSC-derived cells in mitigating inflammation during cartilage regeneration. To fully harness the immunomodulatory capacity of iPSC-derived cells, further studies are warranted to unravel the specific regulatory mechanisms by which these cells modulate inflammation in the joint environment.

Another noteworthy aspect of this study is the utilization of a skeletally mature translational model for chondral defect repair. It has been observed in previous studies that articular cartilage in younger models has a higher capacity for self-regeneration compared to older models [43, 44]. For example, Varasa et al. reported that articular cartilage of skeletally immature pigs retains the ability for spontaneous repair [45]. Their study showed no significant difference in cartilage repair between the autologous chondrocyte implantation group and the sham group in pigs treated at 8 months of age, as evaluated at 3 and 12 months follow-ups. To mitigate the potential confounding effect of spontaneous cartilage repair, which is often observed in immature minipigs, we allowed the animals to reach 24 months of age before performing the experimental surgery. It is noteworthy that the acellular group demonstrated the least favorable outcomes in terms of cartilage repair, aligning with the existing understanding that adult cartilage possesses limited regenerative potential. By employing skeletally mature animals in our experimental design, we aimed to provide a more objective evaluation of cartilage repair that closely resembles the clinical scenario in adult human patients.

Our study demonstrated that iMSC-Chs not only exhibited higher chondrogenic potential in repairing cartilage lesions but also generated hyaline cartilage, as evidenced by the predominant expression of the hyaline cartilage-associated marker COL2A1 and minimal expression of the hypertrophic chondrocyte-associated marker COL10A1. Particularly, the mRNA expression results shown in Fig. 4E, which demonstrate reduced expression of hypertrophic chondrocyte markers such as *MMP13*, *RUNX2*, *COL10A1*, and *ALP* in iMSC-Chs, suggest that iMSCs were specifically induced to differentiate into hyaline cartilage chondrocytes capable of maintaining a stable, non-hypertrophic phenotype prior to implantation in animals. In contrast, BMSCs undergoing chondrogenesis in culture were found to develop a hypertrophic chondrocyte phenotype, revealing a critical distinction in the chondrogenic outcomes between these cell types. These findings align with a recent study by Pothiawala et al., which identified a subpopulation of iPSC derivatives capable of generating and maintaining a “true” permanent-like articular cartilage phenotype that does not progress toward endochondral ossification [46]. They identified a chondroprogenitor population positive for growth differentiation factor 5 derived from iPSC-derived mesenchymal cells along the paraxial mesodermal lineage. Overall, our study demonstrates the potential of iPSC derivatives to generate hyaline-like cartilage suitable for joint defect repair, with the goal of achieving long-term cartilage stability and function.

The current findings are in line with our previous research, demonstrating the superior cartilage repair capacity of human iMSCs compared to their parental MSCs derived from synovial fluid in a spontaneous osteoarthritis model [47]. However, conflicting results from other studies suggest that BMSCs may have higher chondrogenic potential than iPSC derivatives. For example, Diederichs et al. compared iPSC-derived MSC-like progenitor cells (iMPCs) with parental BMSCs and found that BMSCs showed higher expression of cartilage-associated markers and greater GAG production after chondrogenesis induction [48]. The disparity in results could be due to differences in MSC induction procedures, which might lead to the emergence of distinct MSC subpopulations with varying chondrogenic differentiation capacities. Diederichs et al. employed a range of methodologies, including embryoid body outgrowth, spontaneous differentiation, indirect BMSC coculture, and direct induction using BMSC growth medium to generate iMPCs. In contrast, our approach utilized a commercial product with iMSC induction standardized according to the manufacturer’s protocol. This method likely ensures a more uniform cell population, which could account for the enhanced chondrogenic potential we observed. The variance in induction approaches between the studies is a probable cause of the observed differences in chondrogenic outcomes.

We found that TGFB1 alone was sufficient to induce chondrogenesis in both minipig BMSCs and iMSCs during a 21-day culture period. The strategy of using TGFB alone to induce chondrogenesis in iMSCs is supported by the established understanding that this growth factor is a dominant inducer of chondrogenesis in primary MSCs and has been utilized in various studies to compare the chondrogenic capacities of primary MSCs and iMSCs under identical induction conditions [48–50]. However, it is well known that the chondrogenic potential of MSCs is also influenced by the composition of the chondrogenic medium used during differentiation. Different medium formulations can significantly impact chondrogenic outcomes, as cells may require distinct growth factors and signaling molecules for optimal differentiation. For example, primary MSCs often respond effectively to growth factors such as TGFBs, BMPs, FGF2, and IGF1, which are essential for promoting cartilage matrix production and the chondrocyte-like phenotype [51]. In contrast, iMSCs may have unique signaling requirements that necessitate a specific set of growth factors or adjusted concentrations for effective chondrogenesis [50]. Research has suggested that iMSCs might need particular combinations or varying concentrations of growth factors to reach the chondrogenic efficiency of primary MSCs [52]. Additionally, variations in other components of the induction medium can further contribute to the differences in chondrogenic potential observed between these cell types [53], suggesting that a standardized approach may be insufficient for achieving consistent chondrogenic differentiation across different MSC sources. Thus, tailoring the chondrogenic medium for each MSC type could be crucial for maximizing chondrogenic outcomes.

While the potential of iPSC derivatives for cartilage regeneration has been extensively investigated, including in our study, there remains a common limitation of small sample sizes among reported studies. This limitation contributes to variable results observed among animals. Additionally, it is worth noting that BMSCs may not be the optimal cell type for repairing chondral defects due to their inherent propensity towards endochondral ossification. Recent studies have shown promising outcomes by utilizing chondrocytes derived directly from iPSCs, bypassing the MSC stage [54, 55]. This approach potentially avoids the hypertrophy often observed in MSC-derived chondrocytes and may offer a more direct and effective method for generating hyaline cartilage. However, further investigation and comparison between these two strategies are necessary to determine the most suitable approach for successful cartilage regeneration.

In conclusion, this study demonstrates the potential of autologous iPSCs as a viable source of therapeutic cells for repairing articular cartilage defects. We provide evidence that iPSC-derived chondrocytes show comparable cartilaginous matrix production to BMSC-derived chondrocytes while exhibiting higher proliferation rates. Moreover, autologous iMSC-Ch-laden implants promote hyaline cartilage formation and enhance the immune microenvironment of the joint. These findings highlight the promising role of iPSC derivatives in cartilage repair and their potential as a safe and effective therapeutic strategy. Future studies should aim to optimize the chondrogenic potential of iPSC derivatives and investigate their long-term effects in larger animal models to further validate their clinical translatability.

## Electronic supplementary material

Below is the link to the electronic supplementary material.

Supplementary Material 1: Chondrogenic evaluation of iMSCs and BMSCs in pellet culture. Transcript levels of hypertrophic chondrocyte-associated markers (MMP13, RUNX2, COL10A1, and ALP) and fibrocartilage-associated marker (COL1A1 and COL3A1) during 21-day chondrogenic differentiation of iMSCs or BMSCs. *p > 0.05, **p > 0.01; n = 3 biological replicates. N.D.: not detected.

## Data Availability

All data collected or analyzed during this study are included within this published article.

## References

[1] Pizzute T, Lynch K, Pei M. Impact of tissue-specific stem cells on lineage-specific differentiation: a focus on the musculoskeletal system. Stem Cell Rev Rep. 2015;11(1):119–32. 25113801 10.1007/s12015-014-9546-8 PMC4326629

[2] Mithoefer K, McAdams T, Williams RJ, Kreuz PC, Mandelbaum BR. Clinical efficacy of the microfracture technique for articular cartilage repair in the knee: an evidence-based systematic analysis. Am J Sports Med. 2009;37(10):2053–63. 19251676 10.1177/0363546508328414

[3] Saris DB, Vanlauwe J, Victor J, Haspl M, Bohnsack M, Fortems Y, Vandekerckhove B, Almqvist KF, Claes T, Handelberg F, et al. Characterized chondrocyte implantation results in better structural repair when treating symptomatic cartilage defects of the knee in a randomized controlled trial versus microfracture. Am J Sports Med. 2008;36(2):235–46. 18202295 10.1177/0363546507311095

[4] Knutsen G, Drogset JO, Engebretsen L, Grøntvedt T, Isaksen V, Ludvigsen TC, Roberts S, Solheim E, Strand T, Johansen O. A randomized trial comparing autologous chondrocyte implantation with microfracture. Findings at five years. J Bone Joint Surg Am. 2007;89(10):2105–12. 17908884 10.2106/JBJS.G.00003

[5] Hunziker EB. Articular cartilage repair: basic science and clinical progress. A review of the current status and prospects. Osteoarthritis Cartilage. 2002;10(6):432–63. 12056848 10.1053/joca.2002.0801

[6] Futrega K, Robey PG, Klein TJ, Crawford RW, Doran MR. A single day of TGF-β1 exposure activates chondrogenic and hypertrophic differentiation pathways in bone marrow-derived stromal cells. Commun Biol. 2021;4(1):29. 33398032 10.1038/s42003-020-01520-0 PMC7782775

[7] Takahashi K, Yamanaka S. Induction of pluripotent stem cells from mouse embryonic and adult fibroblast cultures by defined factors. Cell. 2006;126(4):663–76. 16904174 10.1016/j.cell.2006.07.024

[8] Jiao H, Walczak BE, Lee MS, Lemieux ME, Li WJ. GATA6 regulates aging of human mesenchymal stem/stromal cells. Stem Cells. 2021;39(1):62–77. 33252174 10.1002/stem.3297 PMC7772271

[9] Lee J, Taylor SE, Smeriglio P, Lai J, Maloney WJ, Yang F, Bhutani N. Early induction of a prechondrogenic population allows efficient generation of stable chondrocytes from human induced pluripotent stem cells. Faseb j. 2015;29(8):3399–410. 25911615 10.1096/fj.14-269720 PMC4511207

[10] Guzzo RM, Gibson J, Xu RH, Lee FY, Drissi H. Efficient differentiation of human iPSC-derived mesenchymal stem cells to chondroprogenitor cells. J Cell Biochem. 2013;114(2):480–90. 22961870 10.1002/jcb.24388

[11] Diederichs S, Klampfleuthner FAM, Moradi B, Richter W. Chondral differentiation of Induced Pluripotent stem cells without Progression into the Endochondral Pathway. Front Cell Dev Biol. 2019;7:270. 31737632 10.3389/fcell.2019.00270 PMC6838640

[12] Osiecka A, Malejczyk J, Moskalewski S. Cartilage transplants in normal and preimmunized mice. Arch Immunol Ther Exp (Warsz). 1990;38(5–6):461–73. 2130808

[13] Kawabe N, Yoshinao M. The repair of full-thickness articular cartilage defects. Immune responses to reparative tissue formed by allogeneic growth plate chondrocyte implants. Clin Orthop Relat Res 1991(268):279–93. 2060220

[14] Yamaga KM, Bolen H, Kimura L, Lance EM. Enhanced chondrocyte destruction by lymphokine-activated killer cells. Possible role in rheumatoid arthritis. Arthritis Rheum. 1993;36(4):500–13. 8457225 10.1002/art.1780360410

[15] Romaniuk A, Malejczyk J, Kubicka U, Hyc A, Olszewski WL, Moskalewski S. Rejection of cartilage formed by transplanted allogeneic chondrocytes: evaluation with monoclonal antibodies. Transpl Immunol. 1995;3(3):251–7. 8581414 10.1016/0966-3274(95)80032-8

[16] Lee MS, Stebbins MJ, Jiao H, Huang HC, Leiferman EM, Walczak BE, Palecek SP, Shusta EV, Li WJ. Comparative evaluation of isogenic mesodermal and ectomesodermal chondrocytes from human iPSCs for cartilage regeneration. Sci Adv. 2021; 7(21). 10.1126/sciadv.abf0907 PMC8133756 34138734

[17] Xu X, Shi D, Liu Y, Yao Y, Dai J, Xu Z, Chen D, Teng H, Jiang Q. In vivo repair of full-thickness cartilage defect with human iPSC-derived mesenchymal progenitor cells in a rabbit model. Exp Ther Med. 2017;14(1):239–45. 28672920 10.3892/etm.2017.4474 PMC5488398

[18] Wu CL, Dicks A, Steward N, Tang R, Katz DB, Choi YR, Guilak F. Single cell transcriptomic analysis of human pluripotent stem cell chondrogenesis. Nat Commun. 2021;12(1):362. 33441552 10.1038/s41467-020-20598-y PMC7806634

[19] Libbin RM, Rivera ME. Regeneration of growth plate cartilage induced in the neonatal rat hindlimb by reamputation. J Orthop Res. 1989;7(5):674–82. 2760739 10.1002/jor.1100070507

[20] Mukoyama S, Sasho T, Akatsu Y, Yamaguchi S, Muramatsu Y, Katsuragi J, Fukawa T, Endo J, Hoshi H, Yamamoto Y, et al. Spontaneous repair of partial thickness linear cartilage injuries in immature rats. Cell Tissue Res. 2015;359(2):513–20. 25407523 10.1007/s00441-014-2041-3

[21] Kwon DJ, Lee YS, Shin D, Won KH, Song KD. Genome analysis of Yucatan miniature pigs to assess their potential as biomedical model animals. Asian-Australas J Anim Sci. 2019;32(2):290–6. 29879811 10.5713/ajas.18.0170 PMC6325393

[22] Uto S, Nishizawa S, Hikita A, Takato T, Hoshi K. Application of induced pluripotent stem cells for cartilage regeneration in CLAWN miniature pig osteochondral replacement model. Regen Ther. 2018;9:58–70. 30525076 10.1016/j.reth.2018.06.003 PMC6222263

[23] Zhu Y, Wu X, Liang Y, Gu H, Song K, Zou X, Zhou G. Repair of cartilage defects in osteoarthritis rats with induced pluripotent stem cell derived chondrocytes. BMC Biotechnol. 2016;16(1):78. 27829414 10.1186/s12896-016-0306-5 PMC5103600

[24] Jiao H, Lee MS, Sivapatham A, Leiferman EM, Li WJ. Epigenetic regulation of BAF60A determines efficiency of miniature swine iPSC generation. Sci Rep. 2022;12(1):9039. 35641537 10.1038/s41598-022-12919-6 PMC9156668

[25] Lee MS, Wang J, Yuan H, Jiao H, Tsai TL, Squire MW, Li WJ. Endothelin-1 differentially directs lineage specification of adipose- and bone marrow-derived mesenchymal stem cells. Faseb j. 2019;33(1):996–1007. 30096039 10.1096/fj.201800614R PMC6355080

[26] Czaplewski SK, Tsai TL, Duenwald-Kuehl SE, Vanderby R Jr., Li WJ. Tenogenic differentiation of human induced pluripotent stem cell-derived mesenchymal stem cells dictated by properties of braided submicron fibrous scaffolds. Biomaterials. 2014;35(25):6907–17. 24875762 10.1016/j.biomaterials.2014.05.006

[27] Hurtig MB, Buschmann MD, Fortier LA, Hoemann CD, Hunziker EB, Jurvelin JS, Mainil-Varlet P, McIlwraith CW, Sah RL, Whiteside RA. Preclinical studies for cartilage repair: recommendations from the International Cartilage Repair Society. Cartilage. 2011;2(2):137–52. 26069576 10.1177/1947603511401905 PMC4300779

[28] Sarin JK, Brommer H, Argüelles D, Puhakka PH, Inkinen SI, Afara IO, Saarakkala S, Töyräs J. Multimodality scoring of chondral injuries in the equine fetlock joint ex vivo. Osteoarthritis Cartilage. 2017;25(5):790–8. 27965140 10.1016/j.joca.2016.12.007

[29] Shihan MH, Novo SG, Le Marchand SJ, Wang Y, Duncan MK. A simple method for quantitating confocal fluorescent images. Biochem Biophys Rep. 2021;25:100916. 33553685 10.1016/j.bbrep.2021.100916 PMC7856428

[30] Bravery CA. Do human leukocyte antigen-typed cellular therapeutics based on induced pluripotent stem cells make commercial sense? Stem Cells Dev. 2015;24(1):1–10. 25244598 10.1089/scd.2014.0136

[31] Eliopoulos N, Stagg J, Lejeune L, Pommey S, Galipeau J. Allogeneic marrow stromal cells are immune rejected by MHC class I- and class II-mismatched recipient mice. Blood. 2005;106(13):4057–65. 16118325 10.1182/blood-2005-03-1004

[32] Sohn EH, Jiao C, Kaalberg E, Cranston C, Mullins RF, Stone EM, Tucker BA. Allogenic iPSC-derived RPE cell transplants induce immune response in pigs: a pilot study. Sci Rep. 2015;5:11791. 26138532 10.1038/srep11791 PMC4490339

[33] Jones M, Symmons D, Finn J, Wolfe F. Does exposure to immunosuppressive therapy increase the 10 year malignancy and mortality risks in rheumatoid arthritis? A matched cohort study. Br J Rheumatol. 1996;35(8):738–45. 8761185 10.1093/rheumatology/35.8.738

[34] Abe K, Yamashita A, Morioka M, Horike N, Takei Y, Koyamatsu S, Okita K, Matsuda S, Tsumaki N. Engraftment of allogeneic iPS cell-derived cartilage organoid in a primate model of articular cartilage defect. Nat Commun. 2023;14(1):804. 36808132 10.1038/s41467-023-36408-0 PMC9941131

[35] Mattapally S, Pawlik KM, Fast VG, Zumaquero E, Lund FE, Randall TD, Townes TM, Zhang J. Human leukocyte Antigen Class I and II knockout Human Induced Pluripotent Stem Cell-Derived cells: Universal Donor for Cell Therapy. J Am Heart Assoc. 2018;7(23):e010239. 30488760 10.1161/JAHA.118.010239 PMC6405542

[36] Deuse T, Hu X, Gravina A, Wang D, Tediashvili G, De C, Thayer WO, Wahl A, Garcia JV, Reichenspurner H, et al. Hypoimmunogenic derivatives of induced pluripotent stem cells evade immune rejection in fully immunocompetent allogeneic recipients. Nat Biotechnol. 2019;37(3):252–8. 30778232 10.1038/s41587-019-0016-3 PMC6419516

[37] Ben-David U, Benvenisty N. The tumorigenicity of human embryonic and induced pluripotent stem cells. Nat Rev Cancer. 2011;11(4):268–77. 21390058 10.1038/nrc3034

[38] Saito T, Yano F, Mori D, Kawata M, Hoshi K, Takato T, Masaki H, Otsu M, Eto K, Nakauchi H, et al. Hyaline cartilage formation and tumorigenesis of implanted tissues derived from human induced pluripotent stem cells. Biomed Res. 2015;36(3):179–86. 26106047 10.2220/biomedres.36.179

[39] Okita K, Matsumura Y, Sato Y, Okada A, Morizane A, Okamoto S, Hong H, Nakagawa M, Tanabe K, Tezuka K, et al. A more efficient method to generate integration-free human iPS cells. Nat Methods. 2011;8(5):409–12. 21460823 10.1038/nmeth.1591

[40] Prieto J, Seo AY, León M, Santacatterina F, Torresano L, Palomino-Schätzlein M, Giménez K, Vallet-Sánchez A, Ponsoda X, Pineda-Lucena A, et al. MYC induces a hybrid energetics Program Early in Cell Reprogramming. Stem Cell Rep. 2018;11(6):1479–92. 10.1016/j.stemcr.2018.10.018 PMC6294174 30472011

[41] Petrigliano FA, Liu NQ, Lee S, Tassey J, Sarkar A, Lin Y, Li L, Yu Y, Geng D, Zhang J, et al. Long-term repair of porcine articular cartilage using cryopreservable, clinically compatible human embryonic stem cell-derived chondrocytes. NPJ Regen Med. 2021;6(1):77. 34815400 10.1038/s41536-021-00187-3 PMC8611001

[42] Chang YH, Wu KC, Ding DC. Induced Pluripotent Stem Cell-Differentiated Chondrocytes Repair Cartilage Defect in a Rabbit Osteoarthritis Model. Stem Cells Int. 2020; 2020:8867349. 10.1155/2020/8867349 PMC7671807 33224204

[43] Namba RS, Meuli M, Sullivan KM, Le AX, Adzick NS. Spontaneous repair of superficial defects in articular cartilage in a fetal lamb model. J Bone Joint Surg Am. 1998;80(1):4–10. 9469302 10.2106/00004623-199801000-00003

[44] Wei X, Messner K. Maturation-dependent durability of spontaneous cartilage repair in rabbit knee joint. J Biomed Mater Res. 1999;46(4):539–48. 10398015 10.1002/(sici)1097-4636(19990915)46:4<539::aid-jbm12>3.0.co;2-s

[45] Vasara AI, Hyttinen MM, Pulliainen O, Lammi MJ, Jurvelin JS, Peterson L, Lindahl A, Helminen HJ, Kiviranta I. Immature porcine knee cartilage lesions show good healing with or without autologous chondrocyte transplantation. Osteoarthritis Cartilage. 2006;14(10):1066–74. 16720098 10.1016/j.joca.2006.04.003

[46] Pothiawala A, Sahbazoglu BE, Ang BK, Matthias N, Pei G, Yan Q, Davis BR, Huard J, Zhao Z, Nakayama N. GDF5 + chondroprogenitors derived from human pluripotent stem cells preferentially form permanent chondrocytes. Development. 2022; 149(11). 10.1242/dev.196220 PMC9245189 35451016

[47] Walczak BE, Jiao H, Lee MS, Li WJ. Reprogrammed synovial fluid-derived mesenchymal Stem/Stromal cells acquire enhanced therapeutic potential for articular cartilage repair. Cartilage. 2021;13(2suppl):s530–43. 10.1177/19476035211040858 PMC8804808 34467773

[48] Diederichs S, Tuan RS. Functional comparison of human-induced pluripotent stem cell-derived mesenchymal cells and bone marrow-derived mesenchymal stromal cells from the same donor. Stem Cells Dev. 2014;23(14):1594–610. 24625206 10.1089/scd.2013.0477 PMC4086513

[49] Wu JY, Yeager K, Tavakol DN, Morsink M, Wang B, Soni RK, Hung CT, Vunjak-Novakovic G. Directed differentiation of human iPSCs into mesenchymal lineages by optogenetic control of TGF-β signaling. Cell Rep. 2023;42(5):112509. 37178118 10.1016/j.celrep.2023.112509 PMC10278972

[50] Xu M, Shaw G, Murphy M, Barry F. Induced Pluripotent Stem cell-derived mesenchymal stromal cells are functionally and genetically different from bone marrow-derived mesenchymal stromal cells. Stem Cells. 2019;37(6):754–65. 30779868 10.1002/stem.2993 PMC6591688

[51] Shestovskaya MV, Bozhkova SA, Sopova JV, Khotin MG, Bozhokin MS. Methods of modification of mesenchymal stem cells and conditions of their culturing for Hyaline Cartilage tissue Engineering. Biomedicines. 2021; 9(11). 10.3390/biomedicines9111666 PMC8615732 34829895

[52] Zujur D, Al-Akashi Z, Nakamura A, Zhao C, Takahashi K, Aritomi S, Theoputra W, Kamiya D, Nakayama K, Ikeya M. Enhanced chondrogenic differentiation of iPS cell-derived mesenchymal stem/stromal cells via neural crest cell induction for hyaline cartilage repair. Front Cell Dev Biol. 2023;11:1140717. 37234772 10.3389/fcell.2023.1140717 PMC10206169

[53] Xiang S, Lin Z, Makarcyzk MJ, Riewruja K, Zhang Y, Zhang X, Li Z, Clark KL, Li E, Liu S, et al. Differences in the intrinsic chondrogenic potential of human mesenchymal stromal cells and iPSC-derived multipotent cells. Clin Transl Med. 2022;12(12):e1112. 36536500 10.1002/ctm2.1112 PMC9763539

[54] Yoshimatsu M, Ohnishi H, Zhao C, Hayashi Y, Kuwata F, Kaba S, Okuyama H, Kawai Y, Hiwatashi N, Kishimoto Y, et al. In vivo regeneration of rat laryngeal cartilage with mesenchymal stem cells derived from human induced pluripotent stem cells via neural crest cells. Stem Cell Res. 2021;52:102233. 33607469 10.1016/j.scr.2021.102233

[55] Adkar SS, Wu CL, Willard VP, Dicks A, Ettyreddy A, Steward N, Bhutani N, Gersbach CA, Guilak F. Step-wise Chondrogenesis of Human Induced Pluripotent Stem cells and Purification Via a reporter Allele Generated by CRISPR-Cas9 Genome Editing. Stem Cells. 2019;37(1):65–76. 30378731 10.1002/stem.2931 PMC6312762

