# Supplementary figures and images for: Autologous iPSC- and MSC-derived chondrocyte implants for cartilage repair in a miniature pig model
Source: Stem Cell Res Ther. 2025 Feb 23;16:86. doi: 10.1186/s13287-025-04215-7 (PMC11849328; doi:10.1186/s13287-025-04215-7)

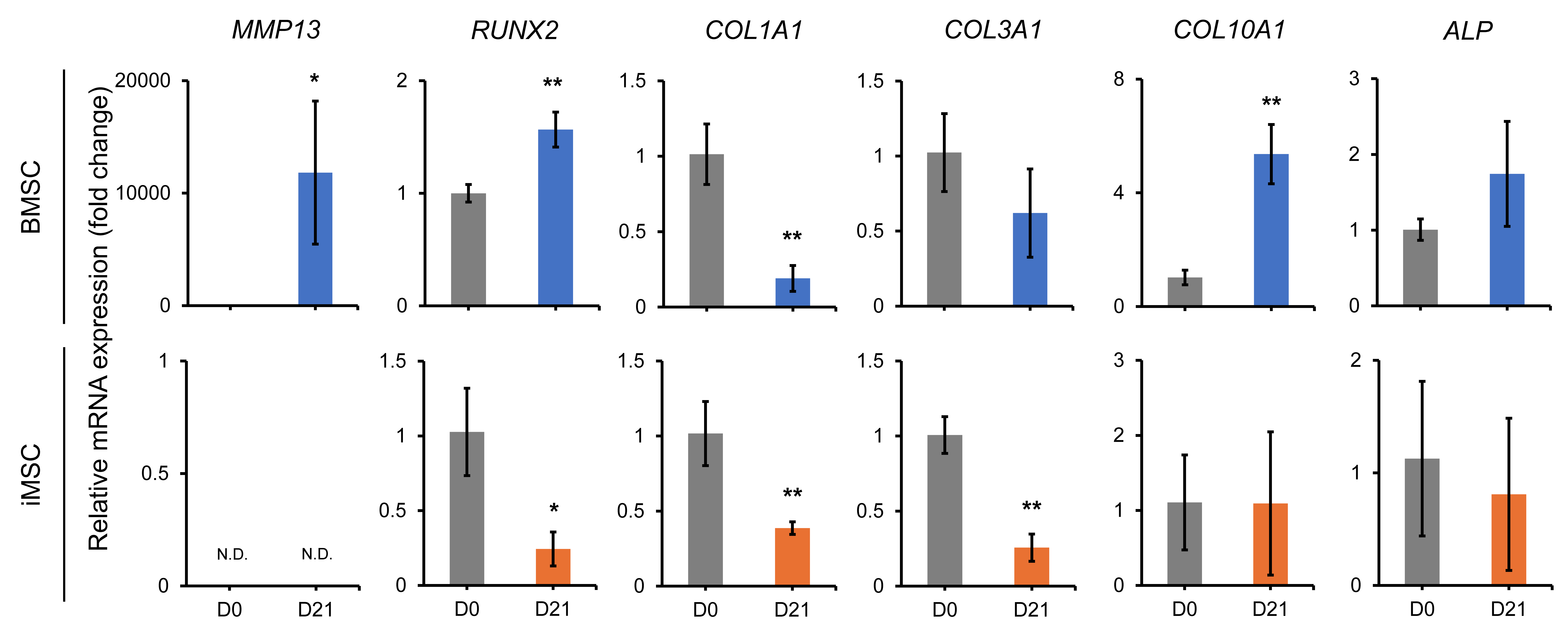

Supplement: Supplementary file 1 — Supplementary Material 1: Chondrogenic evaluation of iMSCs and BMSCs in pellet culture. Transcript levels of hypertrophic chondrocyte-associated markers (MMP13, RUNX2, COL10A1, and ALP) and fibrocartilage-associated marker (COL1A1 and COL3A1) during 21-day chondrogenic differentiation of iMSCs or BMSCs. *p > 0.05, **p > 0.01; n = 3 biological replicates. N.D.: not detected. [file 13287_2025_4215_MOESM1_ESM.tif]
